# Degeneration of the Olfactory Guanylyl Cyclase D Gene during Primate Evolution

**DOI:** 10.1371/journal.pone.0000884

**Published:** 2007-09-12

**Authors:** Janet M. Young, Hang Waters, Cora Dong, Hans-Jürgen Fülle, Emily R. Liman

**Affiliations:** 1 Division of Human Biology, Fred Hutchinson Cancer Research Center, Seattle, Washington, United States of America; 2 Department of Biological Sciences, University of Southern California, Los Angeles, California, United States of America; 3 Departments of Cell and Neurobiology and Ophthalmology, Keck School of Medicine, University of Southern California, Los Angeles, California, United States of America; 4 Program in Neuroscience, University of Southern California, Los Angeles, California, United States of America; The Rockefeller University, United States of America

## Abstract

**Background:**

The mammalian olfactory system consists of several subsystems that detect specific sets of chemical cues and underlie a variety of behavioral responses. Within the main olfactory epithelium at least three distinct types of chemosensory neurons can be defined by their expression of unique sets of signal transduction components. In rodents, one set of neurons expresses the olfactory-specific guanylyl cyclase (GC)-D gene (*Gucy2d*, guanylyl cyclase 2d) and other cell-type specific molecules. GC-D-positive neurons project their axons to a small group of atypical “necklace” glomeruli in the olfactory bulb, some of which are activated in response to suckling in neonatal rodents and to atmospheric CO_2_ in adult mice. Because GC-D is a pseudogene in humans, signaling through this system appears to have been lost at some point in primate evolution.

**Principal Findings:**

Here we used a combination of bioinformatic analysis of trace-archive and genome-assembly data and sequencing of PCR-amplified genomic DNA to determine when during primate evolution the functional gene was lost. Our analysis reveals that GC-D is a pseudogene in a large number of primate species, including apes, Old World and New World monkeys and tarsier. In contrast, the gene appears intact and has evolved under purifying selection in mouse, rat, dog, lemur and bushbaby.

**Conclusions:**

These data suggest that signaling through GC-D-expressing cells was probably compromised more than 40 million years ago, prior to the divergence of New World monkeys from Old World monkeys and apes, and thus cannot be involved in chemosensation in most primates.

## Introduction

The ability to detect chemicals in the environment is vital for many species to survive and reproduce, allowing individuals to locate food, establish a territory, find mates, and avoid predation or other dangers. In most mammals, two olfactory organs detect environmental chemicals: the main olfactory epithelium (MOE), which lines the nasal cavities, and the vomeronasal organ (VNO), a tubular structure which lies beneath the nasal cavity and is accessed by chemicals dissolved in the luminal fluid [1]. The vomeronasal organ has long been thought to contain sensory neurons that respond to pheromones, chemicals released by animals of the same species that elicit stereotyped behavioral and neurendocrine responses, although it has also been recognized that vomeronasal neurons can respond to chemicals that are not strictly defined as pheromones [2], [3]. The main olfactory system contains a number of subsystems, some of which might also be involved in pheromone detection or in the detection of other specific environmental signals [4]–[6]. Interestingly, in humans the vomeronasal organ appears to be vestigial: function was probably lost prior to the divergence of OW monkeys and apes [3], [7]–[9]. Whether one or more of the main olfactory subsystems might function to detect pheromones or other specific environmental signals in humans is not known.

The majority of cells in the MOE express members of the large family of odorant receptors identified by Buck and Axel (1991) [10] and associated downstream signal transduction components. These cells are likely to detect general odorants, including those that animals use to orient towards food. At least three additional subsets of cells can be identified in the nasal epithelium [4]–[6], [11], [12], including a subset that is defined by its expression of the olfactory-specific guanylyl cyclase (GC-D) [13], [14]. Olfactory sensory neurons project their axons to a part of the brain called the olfactory bulb where the axons of cells that express the same odorant receptor converge to form glomeruli [15]. Cells that express GC-D project their axons to an anatomically distinct group of interconnected glomeruli, the necklace glomeruli, that have been implicated in the suckling response of mammals [16], [17] but see [18], and that have been shown recently to respond to atmospheric CO_2_ [19]. Thus it has been suggested that GC-D-expressing olfactory neurons detect a specific subset of chemicals in the environment that might include pheromones [14] and/or CO_2_.

The GC-D protein is encoded by one of seven receptor guanylyl cyclase genes in the mouse genome (GC-A through GC-G); all receptor guanylyl cyclases share a similar topology with an N-terminal extracellular ligand-binding domain, a single transmembrane domain and a C-terminal cytosolic region that contains kinase-like and catalytic domains [20]. By analogy to other receptor guanylyl cyclases, binding of a ligand to the extracellular region of GC-D is likely to promote generation of intracellular cGMP [21]. An elevation of cGMP is predicted to lead to activation of cyclic nucleotide-gated ion channels to generate a depolarizing electrical response [22]. The ligand for one of the best studied membrane guanylyl cyclases, GC-A, is a peptide hormone (atrial natriuretic peptide) [21] and it has been suggested that, similarly, the ligand for GC-D may be a hormone or peptide pheromone [14].

Given the absence of a functional vomeronasal system in Old World monkeys and apes, we wondered whether GC-D expressing cells might mediate pheromone responses in these species. We therefore sought to determine whether a functional GC-D gene is present in a wide range of primate species, by searching a large number of genomic sequences and by sequencing PCR-amplified primate DNA. Our results show that GC-D is a pseudogene in apes, Old World monkeys, New World monkeys and tarsier, indicating that a functional GC-D gene was lost early in primate evolution and that chemical detection in most primate species is unlikely to involve GC-D. Our study also provides one of the first demonstrations of the utility of trace archive sequence data in evolutionary analysis.

## Materials and Methods

### Primate DNA

Genomic DNA was PCR-amplified and sequenced from a number of primate species as described [8], in order to either confirm inactivating changes observed in database sequences or to obtain novel sequence. Primer sequences and PCR conditions are given in Text S1 and Table S1; primers were obtained from Retrogen or IDT DNA Technology. A human BAC containing GC-D, RPCI11-30J7, was obtained, and BAC DNA was purified using a PSI Ψ BAC DNA isolation kit (Princeton Separations, Adelphia, NJ) according to the manufacturer's recommendations. Genomic DNA from rhesus macaque (*Macaca mulatta*) and human (*Homo sapiens*) was obtained from Clontech. Genomic DNA from the following primate species was obtained from the San Diego Zoo: ring-tailed lemur (*Lemur catta*), titi monkey (*Callicebus moloch*), owl monkey (*Aotus azarai*), red-backed squirrel monkey (*Saimiri oerstedii*), common squirrel monkey (*Saimiri sciureus*), pygmy marmoset (*Callithrix pygmaea*), spider monkey (*Ateles geoffroyi*), howler monkey (*Alouatta seniculus*), drill (*Mandrillus leucophaeus*), siamang (*Hylobates syndactylus*), Sumatran orangutan (*Pongo pygmaeus abelii*), western lowland gorilla (*Gorilla gorilla*), common chimpanzee (*Pan troglodytes*) and bonobo (*Pan paniscus*).

### Identification of GC-D from sequence databases

Sequence trace databases from treeshrew (*Tupaia belangeri*), mouse lemur (*Microcebus murinus*), bushbaby (*Otolemur garnettii*), tarsier (*Tarsius syrichta*), common marmoset (*Callithrix jacchus*), orangutan (*Pongo pygmaeus*) and Sumatran orangutan (*Pongo pygmaeus abelii*) were obtained from NCBI, and a series of bioinformatic tools was used to identify and align GC-D orthologs. Full details are provided in Text S1, and a brief description follows. The rat GC-D amino-acid sequence was used as query in sensitive tblastn searches of traces from each species [23]. Chromatograms were obtained for all matching sequences, along with their mate-pairs (sequences from the opposite end of the same genomic subclone). phredPhrap (www.phrap.org) was used to assemble traces into contigs, and further rounds of blast searching and phredPhrap were used to extend contigs. Genewisedb [24] was used to compare all members of the guanylyl cyclase family to the resulting contigs, and genomic scaffold sequences were constructed from any contigs that matched GC-D better than any other GC family member, along with additional contigs identified though mate-pair linkage to GC-D containing contigs, or by similarity to dog GC-D intronic sequence. Multipipmaker [25] was used to align the rat GC-D genomic sequence with the resulting scaffold sequences, as well as GC-D genomic sequences identified in the mouse, dog, macaque, chimpanzee and human genome assemblies, and the tool subalign [25] was used to extract alignments of each GC-D exon. After manual addition of sequences obtained from other primates by PCR, all exon alignments were concatenated to produce the final alignment shown in Figure S1.

Alignments were inspected for inactivating mutations, which were labelled according to their exon (e.g. 9A is the 5′-most mutation in exon 9, etc.) (Table 1, Table S2). Mutation positions are given with respect to the rat GC-D cDNA sequence (L37203), e.g. 1958del44 indicates a 44-bp deletion beginning at position 1958; 1859_TAA indicates that a stop codon has been created beginning at position 1859, and 924insTG indicates that the sequence TG has been inserted after position 924. Sequence traces were carefully inspected for any inactivating mutation that appeared only in trace archive data from a single species (e.g. the 6 mutations observed in tarsier): clear, unambiguous peaks were present in all cases. In Table 1 and Table S2, “Exon deleted” indicates that the exon cannot be found in genomic sequence, and that continuous genome assembly sequence without gaps is available for the entire region between the two flanking exons. Trace archive data provides suggestive evidence that some exons are missing from some primate genomes, but given the incomplete nature of trace data, these findings are not presented.

**Table 1:** Inactivating mutations in GC-D observed in more than one primate species.

**Exon**	2											3	4	5	9		10				11			12			13	15	18	Obs^1^	Tot^2^
**Mutn. code3**	A	C	E	F	J	L	M	N	O	P	Q	B	A	A	A	C	D	F	J	K	A	B	D	A	B	C	B	B	A		
**Prosimian**																															
Tarsier	−4	−	−	−	.	.	?	?	?	?	.	.	?	?	.	.	−	−	−	−	X	−	−	−	−	−	−	−	−	6	6
**New World monkeys**																															
Titi	−	−	−	−	−	−	X	X	X	X	−	.	?	?	.	.	−	−	−	−	−	−	−	.	?	.	.	.	.	8	8
Owl m.^5^	.	+	+	+	.	+	+	+	+	+	.	.	?	?	.	.	−	−	X	−	−	−	−	.	?	.	.	.	.	1	9
Rb. s. m.	−	X	X	X	−	X	X	X	X	X	−	.	?	?	.	.	−	−	X	−	−	X	−	.	?	.	.	.	.	13	13
C. s. m.	.	+	+	+	.	+	+	+	+	+	.	.	?	?	.	.	−	−	X	−	−	X	−	.	?	.	.	.	.	2	10
Common mm.	−	X	X	X	X	X	X	X	X	X	X	.	?	?	−	−	−	−	X	−	X	−	−	.	?	.	.	−	.	19	19
Pygmy mm.	−	X	X	X	X	X	X	X	X	X	X	.	?	?	.	.	−	−	X	−	X	−	−	.	?	.	.	.	.	12	12
Spider m.	−	−	−	X	−	−	X	X	X	X	−	.	?	?	.	.	.	.	?	.	.	.	.	.	?	.	.	.	.	5	5
Howler m.	−	−	−	X	−	X	X	X	X	X	−	.	?	?	.	.	.	.	?	.	.	.	.	.	?	.	.	.	.	7	7
**Catarrhine primates**																															
Drill	+	.	.	.	.	.	?	?	?	?	.	−	+	+	X	−	−	−	−	X	−	−	−	X	?	X	.	.	.	5	8
Rhesus mac.	X	.	.	.	.	.	?	?	?	?	.	−	X	X	X	−	−	−	−	X	−	−	−	X	?	X	−	−	−	17	17
Siamang	+	.	.	.	.	.	?	?	?	?	.	.	+	+	.	X	.	X	−	−	.	−	−	−	X	−	.	.	.	4	7
Orangutan	+	.	.	.	.	.	?	?	?	?	.	−	+	+	−	X	X	−	−	−	−	−	X	−	X	−	X	X	−	6	9
Sumatran or.	+	.	.	.	.	.	?	?	?	?	.	−	+	+	−	X	X	−	−	−	−	−	X	−	X	−	X	X	−	6	9
Gorilla	+	.	.	.	.	.	?	?	?	?	.	−	+	+	−	X	−	X	−	−	−	.	.	.	+	.	.	.	?	2	6
Chimpanzee	X	.	.	.	.	.	?	?	?	?	.	X	X	X	−	X	−	X	−	−	−	−	−	−	X	−	−	−	X	8	8
Bonobo	+	.	.	.	.	.	?	?	?	?	.	X	+	+	−	X	−	X	−	−	−	−	−	−	X	−	.	.	+	5	9
Human	X	.	.	.	.	.	?	?	?	?	.	X	X	X	−	X	−	X	−	−	−	−	−	−	X	−	−	−	X	9	9

1 Obs: Number of inactivating mutations observed, not including exon 19 mutations that probably have minimal effect on GC-D protein.

2 Total: Minimum total number of inactivating mutations (observed plus inferred).

3 Mutation codes: 2A, 4A, 5A, exon deleted; 2C, 184insTAG; 2E, 213del11; 2F, 254del14; 2J, 460insC; 2L, 590_TGA; 2M, 640del1; 2N, 735del1; 2O, 775insG; 2P, 813del1; 2Q, 830del1; 3B, 924insTG; 9A, 1936del5; 9C, 1965del44; 10D, 2142del2; 10F, 2204_TAG; 10J, 2229del1; 10K, 2237_TAG; 11A, 2246_TGA; 11B, 2294del4; 11D, 2327del2; 12A, 2474del10; 12B, 2477del1; 12C, 2503del1; 13B, 2597_TAG; 15B, 2981del1; 18A, 3303del1.

4 Symbols: X; Sequence available, mutation present.

+; Sequence unavailable, inferred by parsimony that mutation is present.

−; Sequence available, mutation not present.

.; Sequence unavailable, inferred by parsimony that mutation is not present.

?; Sequence unavailable, cannot infer whether mutation is present.

5 Species abbreviations: m., monkey; Rb. s., Red-backed squirrel; C. s., Common squirrel; mm., marmoset; mac., macaque; or., orangutan.

### Analysis of selective pressures

For *K_a_/K_s_* analysis, the alignment was manually edited to remain in-frame, and stop codons in primate sequences were replaced by gaps. PAML's codeml algorithm (version 3.15, [26]) was used to estimate *K_a_/K_s_* along each branch of the species trees of interest (codeml parameters: model = 1 fix_omega = 0, cleandata = 1; complete parameter list supplied on request). Only a subset of species were included in each analysis, because missing sequence data (exon deletions and/or absence from available data) meant that the number of codons available for analysis from all species was very low unless some species were removed from the analysis. Codeml was also used to perform a statistical test for non-neutral evolution on each branch (codeml parameters: model = 2, cleandata = 1, omega = 1). For the statistical tests, twice the difference in maximum likelihood between nested codeml runs (where fix_omega = 1 or fix_omega = 0) was compared to a chi-squared distribution with one degree of freedom to obtain an initial p-value, which was then Bonferroni-corrected by multiplication with the number of branches tested for that tree.

## Results

### Human GC-D is a pseudogene

**Figure 1:**
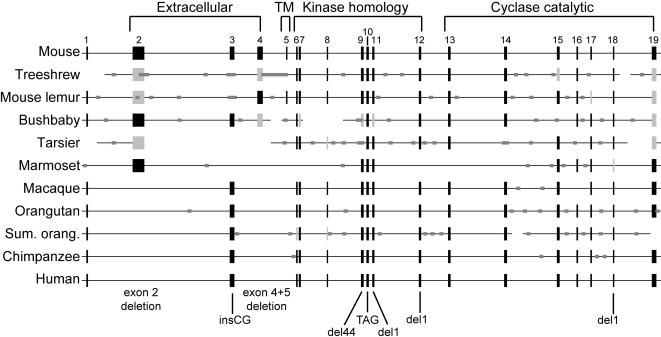
GC-D genomic structures in mouse, treeshrew and various primate genomes, according to genome assemblies (human, chimpanzee, macaque, mouse) or trace archive sequences (other species). Tall black boxes represent exons where full-length, high-quality sequence is available; tall light-gray boxes represent exons where only partial or low-quality sequence is available. Mouse GC-D exons and introns are drawn to scale and numbered (total genomic length 36.8kb), other species are shown with exons aligned to mouse GC-D – in reality, intron sizes differ from mouse. Regions of the protein encoded by each exon are indicated above mouse GC-D (TM: transmembrane). Horizontal lines depict genomic “scaffolds” (see Text S1 – a scaffold consists of a set of multiple “contigs” of overlapping sequence reads, in which the set of contigs are ordered, linked and oriented using paired end sequences); gaps within scaffolds are shown as narrow, dark-gray boxes; gaps between scaffolds are shown as breaks in the horizontal line. Deleterious mutations found in human GC-D are shown below the exons in which they appear: del44 and del1 indicate 44-bp and 1-bp deletions, respectively. Two nonsense mutations and two frameshifts in exon 19 are not shown, as they truncate the protein by only a few amino acids.

Mouse GC-D is encoded by the 19-exon *Gucy2d* gene on chromosome 7E1 (Figure 1). The human ortholog, GUCY2E (Genbank XM_001134425; note this sequence contains errors in exon-intron structure), is located on chromosome 11q13. A publication cataloging all human kinases briefly mentions that human GC-D is a pseudogene [27]; we confirm this finding, showing that human GC-D contains multiple inactivating sequence changes. It should be noted that the human gene whose officially approved name is GUCY2D (Genbank NM_000180) is not the ortholog of mouse *Gucy2d* (GC-D) but instead is the ortholog of the rodent retina-specific *Gucy2e* gene [28](see also MGD www.informatics.jax.org), and is not the gene we discuss here.

Three of the 19 exons present in the mouse GC-D gene (exons 2, 4 and 5) are completely missing from the orthologous human genomic region (Figure 1). In addition, there are ten smaller differences between the human and rodent GC-D genes that disrupt the open reading frame of the human protein (Figure 1, Figure S1, Table S2), including frameshifting insertions and deletions (“indels”), as well as substitutions creating stop codons (nonsense substitutions). Indels and nonsense substitutions occurring in exons 3, 9, 10, 11 and 12 are predicted to generate nonfunctional proteins, which are severely truncated and lack a catalytic domain. In the absence of functional expression data on the intact protein, it is not clear whether the five substitutions/indels in the last two exons (18 and 19) would interfere with function. We sequenced PCR products derived from human genomic DNA in order to confirm that the inactivating changes present in exons 3, 9–12 and 18 are indeed present in the human population and are not merely errors in the human genome assembly.

**Figure 2:**
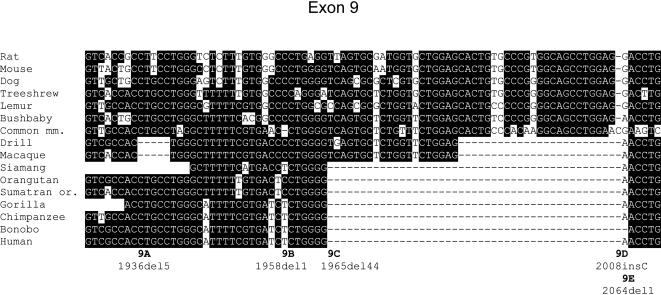
Representative GC-D exon alignment showing multiple inactivating mutations. Alignment of GC-D exon 9 sequences from rat, mouse, dog, treeshrew and various primates. At each alignment position, sequences matching the consensus are shown as white letters with black background, and sequences that do not match are black on white. Insertions/deletions are shown as “−“ characters; areas of missing sequence are entirely blank. Each inactivating mutation is labeled below the alignment: e.g. 9A is the 5′-most mutation in exon 9. Note that the 24-bp deletion in drill and macaque does not introduce a frameshift: it is thus unclear whether it would interfere with function. A full alignment is provided in Figure S1. Species abbreviations: mm., marmoset; or., orangutan.

**Figure 3:**
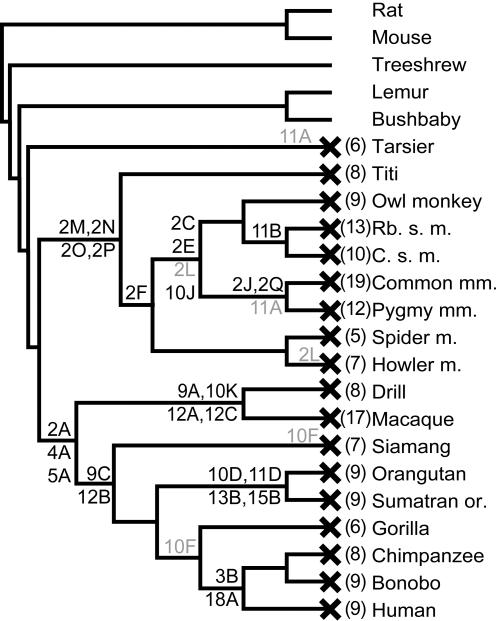
A cladogram of primate species showing approximate appearance time of inactivating mutations. Species relationships were compiled from several sources [47]–[50], and a tree was plotted using MEGA3 (branch lengths not to scale). Inactivating mutations shared by more than one species (Table 1) are marked on the tree (e.g. 9A, etc) at the estimated time point of their appearance. The deletions of exons 2, 4 and 5 are marked as mutations 2A, 4A and 5A. We estimated mutation age conservatively – mutations are marked on the most recent ancestral branch shared by all species possessing the mutation (species known to lack the mutation were used to further pinpoint age). Some mutations may be older than shown, but outgroup data are not available to determine age more accurately. Mutations that appear to have arisen twice independently are marked in gray. Exon 19 mutations are not plotted or included in mutation counts, as their effect on protein sequence may be minimal. An “X” next to a species name indicates that GC-D is a pseudogene in that species, and the number in parentheses indicates the minimum estimate of total number of inactivating mutations (observed plus inferred) present. Species abbreviations: m., monkey; Rb. s., Red-backed squirrel; C. s., Common squirrel; mm., marmoset; or., orangutan.

To establish when during primate evolution GC-D became a pseudogene, we determined whether the GC-D gene is functional in other extant primate species. Two types of data were used: (1) trace archive or genome assembly sequences covering large portions of the GC-D gene, which were available for some species, including representatives of the major divisions of primates: prosimians (mouse lemur, bushbaby and tarsier), New World monkeys (marmoset), Old World monkeys (macaque) and apes (orangutan, Sumatran orangutan and chimpanzee) (Figure 1, Table S3); (2) short sequences obtained by PCR of genomic DNA from a large number of primates. For PCR analysis, we focused on the ∼760-bp exon 2, which is the largest exon of GC-D and thus the most likely to contain deleterious changes, and on exons 3, 9, 10, 11 and 12, which contain deleterious changes in human GC-D. Together these approaches allowed us to identify inactivating changes in a large number of primate species and to deduce likely evolutionary time points at which each inactivating mutation occurred (Table 1, Figure 2, Figure 3).

### GCD was a pseudogene in the common ancestor of apes and Old World monkeys

Examination of the GC-D gene shows that exons 2, 4 and 5 are missing from the genomes of chimpanzee, orangutan, Sumatran orangutan and macaque as well as from human. Because exon 3 is present in these genomes, it can be inferred that two or more events led to the deletion of exons 2, 4 and 5 in the common ancestor of Old World monkeys and apes (collectively known as catarrhine primates). Note that the absence of these exons is highly unlikely to be an artifact of incomplete sequence data: both the macaque and human genome assemblies lack exons 2, 4 and 5 and are gap-free between exons 1 and 14, and there is good coverage of other exons in the chimpanzee and two orangutan genomes. Deletions of exons 2, 4 and 5 remove much of the extracellular and transmembrane domains of the protein and are therefore likely to render it non-functional. In addition, deletion of exons 4 and 5 introduces a frameshift that would likely truncate the protein before the kinase homology and catalytic domains.

The GC-D exons that are still present in catarrhine genomes contain multiple additional inactivating sequence changes (Figure 2, Figure 3, Table 1, Figure S1 and Table S2). We used parsimony to assign each mutation to a branch of the primate tree, and when multiple branch assignments were possible, we conservatively chose the most recent branch that is consistent with available data. Apart from the deletions of exons 2, 4 and 5, no mutation could be unequivocally assigned to the common ancestor of Old World monkeys and apes. Most inactivating mutations are found in just a subset of the catarrhine primates studied, and thus occurred after these species diverged. A frameshift mutation may have been present in the catarrhine ancestor in exon 12, where a 10-bp deletion is present in Old World monkeys at exactly the same genomic position as a 1-bp deletion in apes (Figure 2, mutations 12A and 12B), but it is not possible to distinguish an ancestral 1-bp frameshift from a scenario in which deletions arose at this site twice independently.

The sequence data provides clear evidence that GC-D is a pseudogene in all Old World monkeys and apes. It appears that deletions of exons 2, 4 and 5 occurred in the common ancestor of Old World monkeys and apes or earlier, rendering GC-D non-functional, and that the gene subsequently accumulated additional inactivating changes in the descendant lineages.

### GCD was a pseudogene in the common ancestor of New World monkeys

We also examined trace archive and PCR-product derived sequence data from eight New World monkey species. The GC-D gene of the common marmoset contains at least 19 inactivating mutations. Exon 2 alone contains multiple indels in every species examined (including two nonsense and eight frameshifting indels in marmoset), and in all species the protein product would terminate prematurely within exon 2. Three 1-bp deletions and a 1-bp insertion are present in exon 2 in all species of New World monkeys examined, but absent in lemur and bushbaby, indicating that they originated in the common ancestor of New World monkeys or earlier. Because exon 2 has been deleted from catarrhine genomes, we cannot determine whether the common simian ancestor (the ancestor of New World monkeys and catarrhine primates) also carried the exon 2 mutations shared by all New World monkeys. We did not observe any inactivating mutation shared between New World monkeys and catarrhine primates, and thus cannot determine whether GC-D was already non-functional in the simian ancestor, or whether function was lost independently in the two lineages.

### GC-D is likely intact in lemur and bushbaby, but not in tarsier

Near complete GC-D sequence from three prosimian primates (lemur, bushbaby and tarsier) is available in the trace archive. GC-D is a pseudogene in tarsier, containing several inactivating mutations. Tarsier is the most closely related of these “outgroup” prosimian species to the New World monkeys and catarrhine primates. We found no mutation shared between tarsier and either New World monkeys or catarrhine primates; again, it is not possible to determine whether GC-D function was lost before these species diverged, or lost independently in all three lineages.

In contrast, no inactivating mutations were found in the lemur or bushbaby GC-D sequences, suggesting that the gene is functional in these species. Lemur and bushbaby are “outgroup” species to the New World monkey – catarrhine – tarsier clade (Figure 3). As we have only obtained ∼87% of the coding sequence of lemur GC-D (∼73% for bushbaby), it remains possible that inactivating mutations would be found in the as yet unsequenced portions of these genes. However, because the entire GC-D gene is represented by data from at least one of these two prosimians, we can conclude that the gene was intact in the common ancestor of lemur and bushbaby.

**Figure 4:**
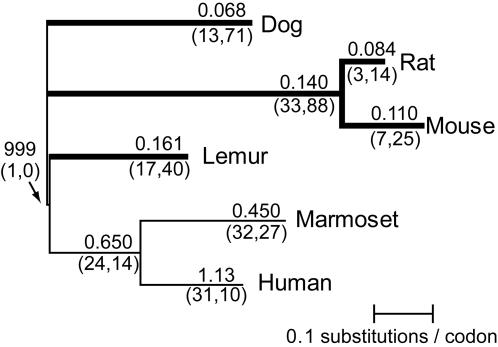
Rodent, dog and lemur GC-D experienced purifying selection; marmoset and human GC-D pseudogenes evolved neutrally. A phylogenetic tree of dog, rat, mouse, lemur, marmoset and human is shown: topology was taken from accepted species trees [47]–[50] and branch lengths represent an estimate of the total number of substitutions per codon in the GC-D sequences examined, as determined by PAML's codeml algorithm. We considered only a subset of species; if all species had been used, missing sequence data (exon deletions and/or absence from available data) would have meant that the number of codons available for analysis was too low. Nonsynonymous (*K_a_*) and synonymous (*K_s_*) rates of evolution were estimated for each branch using PAML's codeml (see Methods, Text S1). The *K_a_/K_s_* ratio is given above each branch, and the number of nonsynonymous and synonymous substitutions, respectively, are given below each branch in parentheses. For each branch of the tree, a statistical test was performed to determine whether the sequences observed are consistent with the null hypothesis of neutral evolution. Branches where the null (neutral) hypothesis was rejected with a Bonferroni-corrected p-value of 0.05 or less (i.e. branches where GC-D evolved under purifying selection) are drawn with thick lines.

To further assess whether GC-D is functional in lemur and bushbaby we examined evolutionary selective pressures by estimating rates of synonymous and nonsynonymous substitutions. For this analysis, we aligned large portions of the gene from multiple primate species and used PAML's codeml algorithm [26] to estimate the ratio of the rates of nonsynonymous and synonymous substitutions (*K_a_/K_s_*) on each branch of the primate species tree (Figure 4 and Figure S2). Synonymous substitutions change the nucleotide sequence but not the amino acid sequence, whereas nonsynonymous substitutions change the amino acid sequence. If a gene is evolving neutrally, the rates of accumulation of synonymous (*K*_s_) and nonsynonymous (*K*_a_) substitutions will be approximately equal, and their ratio (*K*_a_/*K*_s_) will be approximately 1. Under purifying selective pressure, most nonsynonymous substitutions would not be tolerated, and *K*_a_/*K*_s_ will be less than 1. Low *K*_a_/*K*_s_ ratios for a given branch of the tree therefore imply that GC-D was functional for all, or at least a substantial part, of the time represented by that branch of the tree. Statistical tests show that *K*_a_/*K*_s_ is significantly less than 1 on branches leading to bushbaby, lemur, treeshrew, dog, mouse, and rat, indicating that purifying selection acted for much or all of these species' histories. In contrast, GC-D appears to be evolving neutrally on branches leading to a catarrhine primate (human) and a New World monkey (marmoset), as would be expected for a pseudogene.

Thus GC-D appears to have evolved under purifying selection in the lineages leading to lemur, bushbaby, treeshrew, dog, rat and mouse, supporting the idea that GC-D is functional in these species.

## Discussion

Our analysis of primate genomes shows that the gene encoding the olfactory-specific guanylyl cyclase, GC-D, has degenerated to become a pseudogene in humans, other apes, Old World and New World monkeys and tarsier, but appears intact and under purifying selection in lemur and bushbaby. Our findings reinforce a general theme emerging from previous studies that have shown that many components of olfactory and vomeronasal signaling have become pseudogenes over the course of primate evolution. These functional losses appear to have occurred at multiple time points in primate evolution, perhaps corresponding to distinct changes in the ecology of our ancestors [29].

Several lines of evidence suggest that the VNO became vestigial ∼25 million years ago, in the ancestor of Old World monkeys and apes. Inspection of anatomical specimens reveals that the accessory olfactory bulb, the brain region to which VNO neurons project, is absent in Old World monkeys and apes and the VNO in these species, if present, contains only non-sensory cells [3], [7]. Moreover, molecular components of VNO transduction, including all V2R genes and most V1R genes (the vomeronasal receptor gene families) are pseudogenes in Old World monkeys and apes [8], [9], [30]–[34], Young *et al.*, unpublished data]. Deterioration of the VNO can be traced by evolutionary analysis of TRPC2, an ion channel that is critical for VNO function [35]–[38]: TRPC2 is intact in New World monkeys, but acquired inactivating mutations in the ancestor of Old World monkeys and apes ∼25 million years ago [8], [9]. Around the same time, there was a massive loss of functional odorant receptor genes; ∼30% or more of the olfactory receptors (ORs) are pseudogenes in apes and Old World monkeys, compared with only ∼20% of ORs in most New World monkeys, lemur, and mouse [39]–[41]. The acquisition of inactivating mutations in the TRPC2 gene and the olfactory receptor gene family appears to have coincided with the duplication of the red/green opsin gene and acquisition of trichromatic vision, suggesting that visual cues may have in part replaced olfactory and/or pheromonal cues [8], [9], [41].

Additional contraction in the number of functional odorant receptors appears to have occurred more recently in the chimpanzee and human lineages, resulting in a pseudogene proportion of ∼50% for chimp odorant receptors and ∼56% for human odorant receptors [42]. Many of the remaining intact odorant receptor genes show little evidence of selective pressure [40], [43]–[45] and loss of function appears to be ongoing [46]. The basis for this contraction is not known, but may be linked to a change in diet or social behavior.

Our analysis of the GC-D gene shows that it became non-functional early in primate evolution; this event occurred either prior to the divergence of tarsiers, New World monkeys and catarrhine primates, or independently early in each lineage. Rodent GC-D-expressing neurons have been implicated in pheromone detection, and were recently shown to respond to atmospheric CO_2_, possibly providing animals with a means for detecting other individuals at close range [19]. As the function of GC-D-expressing neurons becomes clearer and ligands for rodent GC-D are identified, it will be interesting to know what signaling capacity was lost and to speculate about the ecological changes that could have rendered that signaling unnecessary.

## Supporting Information

Table S1 Oligonucleotide primer sequences
(0.04 MB DOC)

Table S2 All inactivating mutations observed in primate GC-D genes.
(0.05 MB DOC)

Table S3 Sequence datasets used in this study
(0.04 MB DOC)

Figure S1 Alignment of GC-D nucleotide sequences from rat, mouse, dog, treeshrew, and multiple primate species. Evolutionary changes that introduce a frameshift or stop codon that would severely disrupt the protein are highlighted in red; additional frameshifts or stop codons highlighted in gray might have more minimal effects on the protein. The predicted rat GC-D protein sequence is given above each block of the alignment, and below each block, exon boundaries and inactivating mutations are labeled (mutation labels correspond to Supplementary Table S2). Insertions/deletions are shown as “-” characters; areas of missing sequence are entirely blank. Abbreviation: Red-backed squ monkey; red-backed squirrel monkey. The rat cDNA sequence reported by Fülle et al. (L37203) [6] is also given for a small region of exon 2 and for exon 19. Compared to the rat genome assembly, L37203 has a 1-bp insertion and a nearby 1-bp deletion in exon 2, and several 1-bp deletions in exon 19, which together would subtly change the GC-D protein sequence. In all cases, the rat genome assembly sequence appears “correct”, in that it matches GC-D from other species - the discrepancies observed are therefore likely to represent either errors in the cDNA sequence, or polymorphic differences between the rat strain sequenced for the genome project (Brown Norway) and the rat strain from which the cDNA L37203 was derived (Sprague-Dawley).
(0.22 MB DOC)

Figure S2 GC-D evolved under purifying selection in dog, rat, mouse, treeshrew, lemur and bushbaby. A phylogenetic tree of dog, rat, mouse, treeshrew, lemur, and bushbaby is shown: topology was taken from accepted species trees [7]–[10] and branch lengths represent an estimate of the total number of substitutions per codon in the GC-D sequences examined, as determined by PAML's codeml algorithm. We considered only a subset of species; if all species had been used, missing sequence data (exon deletions and/or absence from available data) would have meant that the number of codons available for analysis was too low. Nonsynonymous (Ka) and synonymous (Ks) rates of evolution were estimated for each branch using PAML's codeml (see methods, supplementary methods). The Ka/Ks ratio is given above each branch, and the number of non-synonymous and synonymous substitutions, respectively, are given below each branch in parentheses. For each branch of the tree, a statistical test was performed to determine whether the sequences observed are consistent with the null hypothesis of neutral evolution. Branches where the null (neutral) hypothesis was rejected with a Bonferroni-corrected p-value of 0.05 or less (i.e. branches where GC-D evolved under purifying selection) are drawn with thick lines.
(4.52 MB DOC)

Text S1 (0.06 MB DOC)
