# Supplementary material for: Degeneration of the Olfactory Guanylyl Cyclase D Gene during Primate Evolution
Source: PLoS One. 2007 Sep 12;2(9):e884. doi: 10.1371/journal.pone.0000884 (PMC1964805; doi:10.1371/journal.pone.0000884)
Supplement: Table S1 — Oligonucleotide primer sequences (0.04 MB DOC) [file pone.0000884.s001.doc]

**Table S1. Oligonucleotide primer sequences**

| Exon | Primer | Sequence | Species amplified |
| --- | --- | --- | --- |
| 2 | F1 | GACTRCAKGGGGCTTRTRCCCTGAAG | Titi; pygmy marmoset; howler, red-backed squirrel and spider monkeys |
|  | R1 | CAGGCCRTCCACRCTGCARAGCTGC |
| 9 | F1 | ATGCGGGAGCTGCGGTGTGAG | Drill; rhesus macaque; Sumatran orangutan; bonobo |
|  | R1 | GGTGGTGGTGCAGATACCTCATGCCC |
|  | F2 | ATGCGGGAGCTGCGGTGTGAG | Gorilla |
|  | R2 | CCATGGTCAGTGACCTTCAGCAC |
|  | F3 | TGAGAACGTCGCCACCTGCCTGG | Siamang; chimpanzee; human |
|  | R3 | GGTGGTGGTGCAGATACCTCATGCCC |
| 10/11 | F1 | GGTATCTGCACCACCACCATTTCCC | Drill; siamang; chimpanzee; bonobo; human |
|  | R1 | CTGGAAGTCCCGAGGAGCAGTAG |
|  | F2 | CATTTCCCTCCTGGCTGCCTCAAG | Titi; pygmy mm.[[1]](#footnote-2); owl and red-backed and common squirrel monkeys |
|  | R2 | GTCAGCACCTCCTGCAGGATGATG |
|  | F3 | GGTATCTGCACCACCACCATTTCCC | Sumatran orangutan; gorilla |
|  | R3 | CAGCTCAGGAGCTGTCCATAGCAGC |
| 12 | F1 | AATCATCAGGAGGGTGGCATCTCC | Drill; rhesus macaque; siamang; Sumatran orangutan; bonobo; human |
|  | R1 | CCTGGCTGTAGATCTGGTCCATGC |

1. mm.; marmoset [↑](#footnote-ref-2)
