# Supplementary material for: Degeneration of the Olfactory Guanylyl Cyclase D Gene during Primate Evolution
Source: PLoS One. 2007 Sep 12;2(9):e884. doi: 10.1371/journal.pone.0000884 (PMC1964805; doi:10.1371/journal.pone.0000884)
Supplement: Table S3 — Sequence datasets used in this study (0.04 MB DOC) [file pone.0000884.s003.doc]

**Table S3. Sequence datasets used in this study.**

| Species | Scientific name | Data type | Download date/ assembly version | Number of traces |
| --- | --- | --- | --- | --- |
| Rat | *Rattus norvegicus* | Assembly | rn4 |  |
| Mouse | *Mus musculus* | Assembly | mm8 |  |
| Dog | *Canis familiaris* | Assembly | canFam2 |  |
| Treeshrew | *Tupaia belangeri* | Trace archive | 01/12/07 | 8,611,419 |
| Mouse lemur | *Microcebus murinus* | Trace archive | 01/12/07 | 7,970,179 |
| Bushbaby | *Otolemur garnettii* | Trace archive | 08/02/06 | 8,815,458 |
| Tarsier | *Tarsius syrichta* | Trace archive | 03/13/07 | 14,590,954 |
| Common marmoset | *Callithrix jacchus* | Trace archive | 01/17/07 | 28,216,241 |
| Macaque | *Macaca mulatta* | Assembly | rheMac2 |  |
| Orangutan | *Pongo pygmaeus* | Trace archive | 01/17/07 | 12,157,107 |
| Sumatran orangutan | *Pongo pygmaeus abelii* | Trace archive | 01/17/07 | 16,290,250 |
| Chimpanzee | *Pan troglodytes* | Assembly | panTro2 |  |
| Human | *Homo sapiens* | Assembly | hg18 |  |
