# Supplementary material for: Degeneration of the Olfactory Guanylyl Cyclase D Gene during Primate Evolution
Source: PLoS One. 2007 Sep 12;2(9):e884. doi: 10.1371/journal.pone.0000884 (PMC1964805; doi:10.1371/journal.pone.0000884)
Supplement: Figure S1 — Alignment of GC-D nucleotide sequences from rat, mouse, dog, treeshrew, and multiple primate species. Evolutionary changes that introduce a frameshift or stop codon that would severely disrupt the protein are highlighted in red; additional frameshifts or stop codons highlighted in gray might have more minimal effects on the protein. The predicted rat GC-D protein sequence is given above each block of the alignment, and below each block, exon boundaries and inactivating mutations are labeled (mutation labels correspond to Supplementary Table S2). Insertions/deletions are shown as “-” characters; areas of missing sequence are entirely blank. Abbreviation: Red-backed squ monkey; red-backed squirrel monkey. The rat cDNA sequence reported by Fülle et al. (L37203) [6] is also given for a small region of exon 2 and for exon 19. Compared to the rat genome assembly, L37203 has a 1-bp insertion and a nearby 1-bp deletion in exon 2, and several 1-bp deletions in exon 19, which together would subtly change the GC-D protein sequence. In all cases, the rat genome assembly sequence appears “correct”, in that it matches GC-D from other species - the discrepancies observed are therefore likely to represent either errors in the cDNA sequence, or polymorphic differences between the rat strain sequenced for the genome project (Brown Norway) and the rat strain from which the cDNA L37203 was derived (Sprague-Dawley). (0.22 MB DOC) [file pone.0000884.s004.doc]

95 105 115 125 135

| | | | |

M A G L Q Q G C H P E G Q D W T A

Rat ATGGCAGGTC TGCAGCAGGG CTGTCACCCC GAAGGCCAGG AC-TGGACAG

Mouse ATGGCAGGTC TGCAGCAGGG CTGTCACTTT GAAGGCCAGA AC-TGGACAG

Dog ATAGCAGGAC TAGAGGGAGG CTTGTACCCT GAAGGTTGGT AC-TGGATGC

Treeshrew GAC TGCAGAGGGG CTTGTGCTCC GAAGGCCATG AC-CAGATGC

Mouse lemur ATGGCAG

Ring-tailed lemur GTTGGG AC-TGGATGC

Bushbaby ACAGCAGGAC TACAGGGGGG CTTACCCCCT GAAGATAGGG AC-GGGATGC

Tarsier GAC TGCATGGGGG CTCAAGCCCT GAAGGCGGGT ACCCGGATGC

Titi ATTGGT AC-CGGATGC

Red-backed squ monkey ATTGGT GC-TGGATGC

Common marmoset GAC TGCATGGGAG CTTGTACCCT GAAGATTGGT GC-CAGATGC

Pygmy marmoset attggt gc-cagatgc

Spider monkey ATTTGT GC-CGGATGC

Howler monkey ATTGGT GC-CGGATGC

Macaque GTGGCAG

Orangutan GTGGCAG

Sumatran orangutan GTGGCAG

Chimpanzee GTGGCAG

Human GTTGCAG

ex1 <-||-> ex2 2B

144 154 164 174 183

| | | | |

Rat translated P H W K T C R P C Q G P R G L

Rat cDNA translated A L P G P

Rat CGCCACACTG GAAGACCTGC CGG-CCCTGC CAGGGCCCAC GG---GGACT

Rat cDNA CGCCACACTG GAAGACCTGC CGGGCCCTGC CAGGGCC-AC GG---GGACT

Mouse CACCACACTG GAAGACCTGC CTG-CCCTGC CAGGGCCCGT GG---AGACT

Dog TGCCACATGG GAAGATGTGC CTG-CCTTGC CAGGGCCCAT GG---GGGTC

Treeshrew TGCCACACAG GAAGTCCCGC CTG-CCCTGC CGGGGCCTGT GG---GAACG

Ring-tailed lemur CGCCGTACAG GAAGACCTAC CTG-CTCTGC CAGGGCCCGT GG---GGGTC

Bushbaby TGCTGTACAG GAGGACCACC CTA-CCCTGC CAGGGCCCGT GG---GGTTC

Tarsier TACCGTCCCA GAAGACCTAC CTG-CCCTGC CAGAACCGGC GG---GGCTC

Titi TGCCATACAG GAAGCCCTCC CTG-CCCTGC CAGGGCCCAT GGTGGAGGTC

Red-backed squ monkey TGCTATACAG GAAGCCCTTC CTG-CCCTGC CAGGGCCCAT GGTAGGGGTC

Common marmoset TGCTGTACAG GAAGCCCTCC CTG-CCCTGC CAGGGCCCAT GGTAGGGGTC

Pygmy marmoset tgctgtacag gaagccctcc ctg-ncctgc cagggcccat ggtaggggtc

Spider monkey TGCTGTACAG GAAGCCCTCC CTG-CCCTGC CAGGGCCCAT GGTGGGGGTC

Howler monkey TGCTGTACAG GAAGCCCTCT CTG-CCTTGC CAGGGCCCAT GGTGGGGTTC

2C

190 200 210 220 230

| | | | |

T V R H L R T V S S I S V F S V V

Rat CACGGTCAGA CATCTGAGGA CAGTCTCTTC CATATCGGTC TTCTCTGTGG

Mouse CACTGTCAGT CATCTGAAGA CTGTCTCTTC CATATCAGTC CTCTCTGTGG

Dog CACGTTCAGT CATCTGAGGA CAGCATTTTT CCGTCCTCTT CTCTCTGTGG

Treeshrew CACTGCCGGC CATCCGAGGA GAGCCTCT-- -CTGCCCCTT CTCTCTGGGA

Ring-tailed lemur CACTCTCAGT CATCCAAGGA AAGCCCCTTC CCTGCCACTT CTCTCTGTGG

Bushbaby TACTCTCAGT CATCCAAGGA AAGCCCCTTC CCCACCACTT CTCTCTGTGG

Tarsier CACCCTCAGT CATCCGAGGA AAGCCCCTGA CCTGCCACTT TTCTCTGTGG

Titi TACTCTCAGT CATCCAAAGA CAGCCCCTTT CCTGCCACTC TTCTCTGCGG

Red-backed squ monkey TACTCTCAGT CATCCAAGGA CAG------- ----CCACTC TTCTCTGCGG

Common marmoset TACTCTCAGT CATCCAAGGA CAG------- ----CCACTC TTCTCTGCGG

Pygmy marmoset tactctcagt catccaagga cag------- ----ccactc ttctctgcgg

Spider monkey TACTGTCAGT CATCCAAGGA CAGCCCCTCT CCTGCCACTC TTCTCTGCGG

Howler monkey TACTCTCAGT C-TCCAAGGA CAGCCCTTCT CCTGCCACTC TTCTCTGAGG

2D 2E

240 250 260 270 280

| | | | |

F W G V L L W A D S L S L P A W

Rat TATTTTGGGG TGTCTTGCTG TGGGCTGACT CTCTTTCCCT GCCAGCATGG

Mouse TATTTTGGAG TGTCCTACTT TGGGCTGACT CCCTTTCCCT GTTAGCATGG

Dog TGCTTTGGGG TGCTCTTCTG TGGGCCGGCT CCCTCCCTGA TCTGGCCCAG

Treeshrew TGCTCTGGCC TGCCTTCTTA TGGGCTGGCT TTCTCCCCCA CCTGGTCTGG

Ring-tailed lemur TGCTTTGGGG TGCCCTCCTG GGGGCTGGCT CTGTCCCCCA CCTGGCCTGG

Bushbaby TGGTTAGGGG TGTCCTCCTG TGGGCTGGCT CGCTCTCCCA AGCAGCCTGG

Tarsier TGCTGTGGAG TGCCCTCCTG TGGGTCAGCT CCCTCCCCCA TCTGACCCAG

Titi TGCTTTGGGG TGCCCTCCTG GGGTCTGGCT CCGTCCCACA GCTGGCCTGC

Red-backed squ monkey TGCTTTGGGG TGCC------ --------CT CCCTCCCACA CCTGGCCTGC

Common marmoset TGCTTTGGGA TGCC------ --------CT CCCTCCCACA GCAGGCCTGT

Pygmy marmoset tgctttggga tgcc------ --------ct ccctcccaca gcaggcctgt

Spider monkey TGCTTTGGGG TGCC------ --------CT CCCTCCCACA GCTGGCCTGC

Howler monkey TGCTTTGGGG TGCC------ --------CT CCTTCCCACA GCTGGCCTGC

2F

290 300 310 320 330

| | | | |

A R E T F T L G V L G P W D C D P

Rat GCAAGAGAGA CCTTCACCCT TGGGGTGCTG GGTCCCTGGG ACTGTGACCC

Mouse GCAAGAGAGA CCTTCACCCT TGGGGTGCTG GGTCCCTGGG ACTGTGACCC

Dog GGGGCAGGGA CCTTCACCTT AGGGGTGCTG GGTCCCTGGG ACTGTGACCC

Treeshrew GGGACAGGGA CG

Ring-tailed lemur GGGGCAGGGA CCTTCACCCT GGGGGTGCTG GGTCCCTGGG ACTGTGACGC

Bushbaby GGAGCAGAGA CCTTCACTAT AGCGGTGCTG GGTCCCTGGG ACTGTGATGC

Tarsier GGGGCAAGGA CCTTCATCCT GAGGGTGCTG GGT

Titi AGGGCAGGGA CCTTCACCCT GGTGGTGTTG GGTCCCTGGG ACTGTGACTT

Red-backed squ monkey GGGGCAGGGA CCTTCAT-CT GGTGGTGCTG GGTCCCTGGG ACTGTGACTC

Common marmoset GGGGCAGGGT CCTTCACCCT GATGGTACTG GGTCCCT--- ---------C

Pygmy marmoset ggggcagggt ccttcaccct gatggtactg ggtccct--- ---------c

Spider monkey GGGGCAGGGA CCTTCACCCT GGTGGTGCTG GGTCCCTGGG ACTGTGACTC

Howler monkey AGGGCAGGGA CCTTCACCCT GGTGGTTCTG GGTCCCTGGG ACTGTGACTC

2G

340 350 360 370 380

| | | | |

I F A Q A L P S M A T Q L A V D R

Rat TATCTTTGCC CAGGCACTCC CTAGCATGGC TACCCAGTTG GCTGTAGATC

Mouse CATCTTTGCC CAGGCCCTCC CCAGCATAGC TACCCAGCTG GCTGTAGATC

Dog CATCTTTGCC CAGGCCCTCC CCAGTGTGGC TGCCCAACTG GCTGTGGACC

Ring-tailed lemur CATCTTTGCC CGGGCCCTTC CCAGCTTGGC TGCCCAGCTG GCTGTGGACC

Bushbaby CATCTTTGCC CAGGCCCTCC CCAGAGTGGC TGCCCAGCTA GCTGTGGACC

Titi CGCCTTTGCC CGGGCCTTCC CCTGCGTGGT CACCTAGCTG GTCATGGACC

Red-backed squ monkey CACCTTTGCC CAGGCCTTCC CCTGCGTGGC CACCCAGCTG ACCATGGACC

Common marmoset CACCTTTGCC CAGGTCTTCC CCTGCGTGGC CACACAGCTG ACCATGGACC

Pygmy marmoset cacctttgcc caggtcttcc cctgcgtggc cacacagctg accatggacc

Spider monkey CACCTTTGCC AGGGCCTTCC CCTGCGTGGC CATCCAGCTG ACCATGGACC

Howler monkey CACCTTTGCC AGGGCCTTCC CCTGCATGGC CACCCAGCTG ACCATGGACC

2H

390 400 410 420 430

| | | | |

V N Q D A S L L L G S Q L D F K

Rat GAGTCAATCA GGACGCCTCA CTGCTGCTGG GCTCACAGTT GGATTTCAAG

Mouse AAGTTAATCA GGATGCCTCA CTGCTGCCAG GCTCACAGTT GGATTTCAAG

Dog AAGCCAACCA GGACTCCTCC CTGGTGCTGG GCTCAAGGCT GGTTTCCGTG

Ring-tailed lemur GAACCAAGGG GTACTCCTCA CTGTTGCTGG GCTCACGGCT GGTTTCCGCG

Bushbaby AAGCCAACCA GGACCCCTCA CTGTTGCTGA GCTCGCGGCT GGCTTCTGTG

Titi AATCCAACCA GGACCAGTCA CTGCTGCTGG -CTCACGGCT GGCTTCCGTG

Red-backed squ monkey AATTCAACCA GGACCCGTCA CTGCTGCCGG GCTCACGNCT GGCTTCTGTG

Common marmoset AATCCAACCA GGACCTATCA CTGCTGCTGG GCTCATGGCT GGCTTCCGTG

Pygmy marmoset aatccaacca ggacctatca ctgctgctgg gctcatggct ggcttccgtg

Spider monkey AGTCCAACCA GGACCCATCA CTGCTGCTGG GCTCACGGCT GGCTTCCATG

Howler monkey AATCCAACCA GGACCCAGCA CTGCTGCTGG GCTCACGGCT GGCTTCCATG

2I

440 450 460 469 479

| | | | |

I L P T G C D T P H A L A T F V A

Rat ATCCTCCCTA CAGGCTGTGA C-ACCCCTCA TGCCCTGGCC ACATTTGTGG

Mouse GTCCTCCCCA CAGGCTGTGA C-ACTCCTCA TGCCCTGGCC ACATTCGTGG

Dog GTCCTTCCCA CAGGCTGTGA C-ACCCCTCA CGCCCTGGCC ACATTTCTGG

Ring-tailed lemur GTCCTCCCCA CAGGCTGTGA C-ACCCCTCA CGCCCTGGCC ACATTCCTGG

Bushbaby GTCCTCCCCA CTGCCTGTGA C-ACCCCTCA TGCCCTGGCC ACATTCCTGG

Titi GTCCTCCCTA CAGGCTGTGA C-CCCCCTCC TTCCCTGGCC ACATTCCTGG

Red-backed squ monkey GCCCTCCATA CTGGCTCTGA T-CCCCCTCC TGCCCTGGCC ACATTCCTGG

Common marmoset GTCCTCCCTA CAGGCTGTGA CCCCCCCTCC TGCCCTGGCC ACATTCCTGA

Pygmy marmoset gtcctcccta caggctgtga ccccccctcc tgccctggcc acattcctga

Spider monkey GTCCTCCCTA CAGGCTGTGA C-CCCCCTCC TGCCCTGGCC ACATTCCTGG

Howler monkey GTCCTCCCTA CAGGCTGTGA C-CCCCTTCC TGCCCTGGCC ACATTCCTGG

2J

489 499 509 519 529

| | | | |

H R N T V A A F I G P V N P G Y

Rat CCCACAGGAA CACAGTGGCT GCTTTTATAG GCCCTGTCAA TCCTGGGTAT

Mouse CCCACAAGAA CATAGTGGCT GCTTTTGTAG GCCCTGTTAA TCCTGGGTTC

Dog CCCACAAGAA TACCATAGCT GCTTTTGTGG GTCCCGTCAA TCCTGGTTAC

Ring-tailed lemur CCCACAAGAA CACCGTAGCT GCTTTTGTGG GCCCTGTCAA TCCTGGCTAC

Bushbaby CCCACAGGAA CACCGTAGCT GCTTTTGTGG GCCCTGTCAA TCCTGGCTAC

Titi CCCACAAGAA CACTGTCACT GCTTTCGTGG GCCCTGTCAA TCCTGGCTAC

Red-backed squ monkey CCCACAAGAA CACTGTCACT GCTTTTGTGG GCTCTGTCAA TCCTGGCTAC

Common marmoset CCCACAAGAA CACTGTCACT GCTTTCGTGG GCCCTGTCAA TCCTGGCTAC

Pygmy marmoset cccacaagaa cactgtcact gctttcgtgg gccctgtcaa tcctggctac

Spider monkey CCCACAAGAA CACTGTCACT GCTTCCATGG GCCCTGTCAA TCCTGGCTAC

Howler monkey CCCACAAGAA CACTGTCACT GCTTTCATGG GCCCTGTCAA TCCTGGCTAC

539 549 559 569 579

| | | | |

C P A A A L L A Q G W G K S L F S

Rat TGTCCAGCAG CAGCTCTGCT GGCCCAAGGC TGGGGCAAGT CCCTCTTCTC

Mouse TGCTCAGCAG CAGCCCTGCT GGCCCAAGGC TGGGGCAAGT CCCTCTTCTC

Dog TGCCAAGCAG CAGCCCTGCT GGCCCAAGGC TGGGGCAAGA CCCTCTTCTC

Ring-tailed lemur TGCCCAGCGG CAGCCCTGCT GGCCCAAAGC TGGGGCAAGA CCCTCTTCTC

Bushbaby TGCCCAGCGG CAGCCCTGCT GGCTCAAAGC TGGGGCAAGA CCCTCTTTTC

Titi TGCCCAGCAG CAATCCTGCA GGTCAAAAGC TGGGGCAACA CCCTCTTCTC

Red-backed squ monkey TGCCC----- ------TGCT GGTCAAAATC TGGGGCAACA CCCTCTTCTC

Common marmoset TGCCCAGCAG CAGCCCTGCT GGTCAAAAAC TGGGGCAACA CCCTCTTCTC

Pygmy marmoset tgcccagcag cagccctgct ggtcaaaanc tggggcaaca ccctcttctc

Spider monkey TGCCCAGCAG CAGTCCTGTT GGTCAAAAGC TGTGGCAACA CCCTCTTCTC

Howler monkey TGCCTGGCAG CAGTCCTGTT GGTCAAAAGC TGCGGCAACA CCCTCTTCTC

2K

589 599 609 619 629

| | | | |

W A C G A P E G G G A L V P T L P

Rat CTGGGCCTGT GGAGCTCCAG AGGGAGGAGG TGCCTTAGTG CCCACTTTGC

Mouse CTGGGCCTGT GAAGCTCCAG AGGGAGGAGG TGACTTAGTG CCCACTTTAC

Dog TTGGGCGTGC GGAGCTCCGG AAGGAGGAAG TGAGCTGGTG CCCACCTTGC

Ring-tailed lemur CTGGGCGTGT GGAGTTCCGG AGGGAGGAGG TGAACTGGCA CCCACCTTAC

Bushbaby CTGGGCATGT GGTGCTCCAG AGGGAGGAGG AGAATTGGCA CTCACCTTGC

Titi CTGGGCATGT GGGGCGTCAG AGAGAGGAAG TGAATTGGTG CCCACCTTGC

Red-backed squ monkey CTGAGCATGT GGGGTGTCAG AGAGAGAAGG TGAATTGGTG CCCACCTTGC

Common marmoset CTGAGCCTGT GAGGCATCAG AGAGAGGAGG TGAATTGGTG CCCACCTTGC

Pygmy marmoset ctgagcctgt gaggcatcag agagaggagg tgaattggtg cccaccttgc

Spider monkey CTGGGCATGT GGGGCGTCAG AGAGAGGAGG TGAATTGGTG CCCACCTTGC

Howler monkey CTGAGCATGT GGGGCGTCAG AGAGAGGAGG TGAATTAGTG CCCACCTTGC

2L

639 649 659 669 679

| | | | |

S M A D V L L S V M R H F G W A

Rat CTTCTATGGC CGACGTGCTA CTGTCTGTCA TGAGACACTT TGGCTGGGCT

Mouse CTTCTGCGGC CGATGTGCTA CTGTCTGTCA TGAGACACTT TGGCTGGGCT

Dog CTTCTGCTGC CCATGTGCTG CTGTCTATCA TGAGACACTT TGGCTGGGCT

Ring-tailed lemur CTTCCGCTGT CCACGTGCTG CTGTCCATCA TGAGACACTT TGGCTGGGCT

Bushbaby CTTCTGCTGC CCAGGTGCTG CTGTCCATCA TTAGATACTT CGGCTGGGCT

Titi A-TCTGCTGC CCACGTGCTG CTGTCCATTA TGAGACACTT TGGCTGGGCT

Red-backed squ monkey C-TCTGCTGC CCACGTGCTG CTGTCCATCA TGAGACATTT TGGCTGGGCT

Common marmoset A-TCTGCTGC CCACGTGCTA CTGTTTATCA TGAGACACTT TGGCTGGGCT

Pygmy marmoset a-tctgctgc ccacgtgcta ctgtttatca tgagacactt tggctgggct

Spider monkey A-TCTGCTGC CCACGTGCTG CTGTCCATCA TGAGACACTT TGGCCGGGCT

Howler monkey A-TCTGCTGC CCACGTGCTG CTGTCCATCA TGAGACACTT TGGCTGGGCT

2M

689 699 709 719 729

| | | | |

R L A I V S S H Q D I W V T T A Q

Rat CGCTTGGCCA TCGTGTCCTC TCACCAGGAC ATCTGGGTAA CCACAGCCCA

Mouse CGCTGGGCCA TTGTGTCCTC TCACCAGGAC ATCTGGGTAA CCACAGCCCA

Dog CACGTGGCCA TTGTGTCCTC CCACCAGGAC ATCTGGGTGG CCACAGCCCG

Ring-tailed lemur CGCTCGGCCA TTGTGTCCTC CCACCAGGAC ACCTGGGTGG CCACAGCCCA

Bushbaby CGCCTGGCCA TTGTATCCTC TCACCAGGAC ACCTGGGTGG CCACAGCCCA

Titi CGCTCAGCCA TTGTGTCCTC CCACCAGGAC ACCTGGATGA CCACAG-CCA

Red-backed squ monkey CGCTCAGCCA TTGTGTCCTC CCACCAGGAC ACCTGGATGA CCACAG-CTG

Common marmoset CGCTCAGCCA TTGTGTCCTC CCACCAGGAC ACCTGGATGA CCACGG-CCG

Pygmy marmoset cgctcagcca ttgtgtcctc ccaccaggac acctggatga ccacgg-ccg

Spider monkey TGCTCAGCCA TTGTGTCCTC CCACCAGGAC ACCTGGATGA CCACAG-CCG

Howler monkey CGCTCAGCCA TTGTGTCCTC CCACCAGGAC ACCTGGATGA CCACAG-CCG

2N

739 749 759 769 778

| | | | |

Q L A T A F R A H G L P I G L I

Rat GCAGCTAGCC ACAGCTTTCA GGGCCCATGG GCTGCCT-AT CGGACTGATC

Mouse GCAGCTGGCC ACTGCTTTCA GGACTCATGG GCTGCCC-AT CGGACTGGTG

Dog GCAGGTGGCC ATGACTCTCA GGACACATGG GCTGCCT-GT GGGGTTGGTG

Ring-tailed lemur GCAGCTGGCC ACAGCTTTCA GGACACACNG GCTGCCT-GT GGGGCTGGTA

Bushbaby ACAGCTGGCC ACAACCTTCA GGATCCATGG GCTGCCT-GT GGGGCTGGTG

Titi ACAGCTGGCC ATGACTTTCA GGATACATGG GCTGCGTGGT GGGGCTAGGC

Red-backed squ monkey GCAGCTGGCC GTGGCTTTCA GGGTACATGG GCTGCCTGGT GGGGCTAGGC

Common marmoset GCAGCTGGCC ATGACTTTCA AGAGACATGG GCCGCCTGGT GGGGCTAGGC

Pygmy marmoset gcagctggcc atgactttca agagacatgg gccgcctggt ggggctaggc

Spider monkey GCAGCTGGCC ATGACTTTCA GGATACATGG GCTGCCTGGT GGGGCTAGGC

Howler monkey GCAGCTGGCC ATGACTTTCA GGATACATGG GCTGCCTGGT GGGGCTAGGC

2O

788 795 805 815 825

| | | | |

T S L G P G E K G A T E V C K Q

Rat ---ACCTCCT TGGGACCTGG AGAGAAGGGG GCCACGGAGG TTTGCAAGCA

Mouse ---ACCTCTT TGGGACCTGG AGAGAAGGGG GCCACAGAGG TTTGCAAGCA

Dog ---ACCTCTC TAGGACCTGG AGAGCAGGGG GCCACGGAGG TCCTGGAGCA

Ring-tailed lemur ---ACCTCTT TGGGACCTGG GGAGCAGGGG CCCGAGGAGG TCCCGAA

Bushbaby ---ACCTCTT TGGGACCTGG GGAACAGGGG GCTGTGGAGG TCCTGAAGCA

Titi ACCACCTCTT TGGGACCCAG GAAGCAGG-G GCCACAGAAA TCCCGAA

Red-backed squ monkey GCCACCTCTT TGGGACCGGG GAAGCAGA-G GCCGCAGAAA TCCTGAA

Common marmoset ACCACCTCTT TGGGACCCGG GAAGCAGG-G GCTGCAGAAA TCCCT-AGCA

Pygmy marmoset accacctctt tgggacccgg gaagcagg-g gctgcagaaa tccct-ag

Spider monkey ACCACCTCTT TGGGACCCAG GAAGCAGG-G GCCACAGAAA TACTGAA

Howler monkey ACCACCTCTT TGGGACCCAG GAAGCAGG-G GCCACAGATA TACTGAA

2P 2Q

835 845 855 865 875

| | | | |

L H S V H G L K I V V L C M H S A

Rat GCTCCACAGT GTGCATGGTC TGAAAA

Rat cDNA TCGT GGTTCTGTGC ATGCACTCAG

Mouse GCTCCACAGT GTTCATGGTC TGAAAATTGT GGTTCTGTGC ATGCACTCGG

Dog GCTCTGCAGC GTGGATGGCC TGAAAATTGT GGTGCTGTGC ATGCACTCGG

Bushbaby GCTCTGCAGC GTGGACGGCC TGAAACTTGT GGTGCTGTGC ATGCACTCAG

Common marmoset GCTTTGCAGT GTGGATGGCC TGAAAA

Macaque TTGT GGTGCT-TGG ATGCACTTGG

Orangutan TTGT GGTGCTGTGG ATGCACTTGG

Sumatran orangutan TTGT GGTGCTGTGG ATGCACTTGG

Chimpanzee TTGT GGTGCTGTGG ATGCACTCGG

Human TTGT GGTGCTGTGG ATGCACTCGG

ex2 <-||-> ex3 3A

885 895 905 915 925

| | | | |

L L G G L E Q T V L L R C A R E

Rat cDNA CGCTGCTTGG AGGCCTAGAG CAGACAGTCC TGCTGCGCTG --CGCGAGGG

Mouse CGCTGCTTGG AGGCCTGGAG CAGACCACCC TGCTGCACTG --TGCCTGGG

Dog CGCTCCTGGG GGGCTTGGAG CAGACTGCCC TGCTAAGCCG --AGCGTGGG

Bushbaby CGCTGTTGGG AGGCTCAGAG CAGACCGCCC TGCTGAGCCA --TGCATGGG

Drill CTGAGCCG --TGCCTGGG

Macaque CAGTGCTAGC GGGCTTGGAG ATGACCACCC TGCTGAGCCG --TGCCTGGG

Orangutan CAGTGCTGGG GGGCTTGGAG ATGACCACCC TGCTGAGCCG --TGCCTGGG

Sumatran orangutan CAGTGCTGGG GGGCTTGGAG ATGACCACCC TGCTGAGCCG --TGCCTGGG

Gorilla GGTGCTGGG GGGATTGGAG ATGACCACCC TGCTGAGCCG --TGCCTGGG

Chimpanzee CGGTGCTGGG GGGCTTGGAG ATGACCACCC TGCTGAGCCG TGTGCCTGGG

Bonobo TGCTGAGCCG TGTGCCTGGG

Human CGGTGCTGGG GGGCTTGGAG ATGACCACCC TGCTGAGCCG TGTGCCTGGG

3B

933 943 953 963 973

| | | | |

E G L T D G R L V F L P Y D T L

Rat cDNA AGGAGGGCCT GACAGATGGC AGACTGGTCT TCTTGCCCTA CGACACGCTG

Mouse AGGAGGGCCT CACAGACGGA AGGCTGGTCT TCCTGCCCTA CGACACGCTG

Dog CCCAGGGCCT AGCAGATGGG AGGCTGGTCT TCCTGCCCTA CGATACCATG

Bushbaby CTGAAGGCCT GGCAGACGGG AGGGTGGTCT TCCTGCCCTA CGACACGCTG

Drill CTGAGGGCCT GATGGATGGG AGATTG

Macaque CTGAGGGCCT GATGGATGGG AGATTGGTCT TCCTGCCCTA TGACACGCTG

Orangutan CCAAGGGCCT GGTGGATGGG AGATTAGTCT TCCTGCCCTA TGACACGCTG

Sumatran orangutan CCAAGGGCCT GGTGGATGGG AGATTAGTCT TCCTGCCCTA TGACACGCTG

Gorilla CCGAGGGCCT GGTGGATGGG AGATTA

Chimpanzee CCGAGGGCCT GGTGGATGGG AGATTAGTCT TCCTGCCCTA TGACATGCTG

Bonobo CCGAGGGCCT GGTGGATGGG AGATTA

Human CCGAGGGCCT GGTGGATGGG AGATTAGTCT TCCTGCCCTA TGACATGCTG

983 993 1003 1013 1023

| | | | |

L F A L P Y R N R S Y L V L D D D

Rat cDNA CTCTTTGCCC TGCCCTATCG TAACCGCTCT TACCTGGTCC TGGATGATGA

Mouse CTCTTTGCCC TGCCCTATGG AAACCGCTCC TACCTGGTCC TGGATGACCA

Dog CTCTTCGCCT TGCCCTACCG CAACCACTCC TACCTGGCCC TTGGCAACAG

Bushbaby CTTTTTGCCC TGCCCTACCG CAACCGCTCC TACCCAGCCC TGGGTGCTGG

Macaque CTCTTCTCCC TGCCTTACTG CAACCACTCC TACCTGTCTC TCAGCACTGG

Orangutan CTCTTCTCCC TGTCCTACTG CAACCGCTCC TACCTGTCTC TCTGCACTGG

Sumatran orangutan CTCTTCTCCC TGTCCTACTG CAACCGCTCC TACCTGTCTC TCTGCACTGG

Chimpanzee CTCTTCTCCC TGTCCTACTG CAACTGCTCC TACCTGTCTC TCGGCACTGG

Human CTCTTCTCCC TGTCCTACTG CAACCGCTCC TACCTGTCTC TCGGCACTGG

1033 1043 1053 1063 1072

| | | | |

G P L Q E A Y D A V L T I S L D

Rat cDNA TGGGCCCCTT CAGGAGGCCT ACGATGCAGT ACTCACC-AT CAGTCTGGAC

Mouse TGGGCCCCTT CAGGAAGCCT ATGACGCAGT ACTCACT-GT CAGTCTGGAG

Dog TGGGCCCCTG CAGGAGGTCT ATGATGCAGT CCTCACC-AT CAGCCTGGAG

Bushbaby TGGGCCCCTG CAGAAGGCCT ATGATGCAGT GCTCACC-AT CAGCCTGGAA

Macaque TGGGCCCCCG TGGGAGGCCT ATGATGCAAT GCTCACCCTT CAGCCTAGAG

Orangutan TGGACCCCTG CAGGAGGCCT ATGATGCAGT GCTCACC-AT CAGCCTGGAG

Sumatran orangutan TGGACCCCTG CAGGAGGCCT ATGATGCAGT GCTCACC-AT CAGCCTGGAG

Chimpanzee TGGACCCCTG CAGGAGGCCT ATGATGCAGT GCTCACC-AT CAGCCAGGAG

Human TGGACCCCTG CAGGAGGCCT ATGATGCAGT GCTCACC-AT CAGCCAGGAG

3C

1082 1092 1102 1112 1122

| | | | |

T S P E S H A F T A T K M R G G A

Rat cDNA ACCAGTCCTG AGAGCCATGC CTTCACTGCT ACCAAGATGA GAGGAGGGGC

Mouse TCCAGTCCTG AGAGCCACGC CTTCACTGCT ACCGAGATGA GTGGAGGGGC

Dog TCTGACCCTG TGGACAAGGC CTTTGAAGCT GCCAAGGCCA GTGGAGAAGT

Bushbaby TCCAGCCCTG CAGAGGATGC CTTTACTGCT GCCAGGGTGG GTGGAGAGAT

Macaque TCTGGCCCCA TAGACAAGGC CTTTGCTGCT GCCTGGCCCG ATGGAGAGGC

Orangutan TCTGGCCCCA TAGACAAGGC CTTTGCTGCT GCCCGGCCCA GTGGAGAGGT

Sumatran orangutan TCTGGCCCCA TAGACAAGGC CTTTGCTGCT GCCCGGCCCA GTGGAGAGGT

Chimpanzee TCTGGCCCCA TAGACAAGGC CTTTGCTGCT GCCCTGCCCG ATGGAGAGGT

Human TCTGGCCCCA TAGACAAGGC CTTTGCTGCT GCCCGGCCTG ATGGAGAGGC

1132 1142 1152 1162 1172

| | | | |

A A N L G P E Q V S P L F G T I Y

Rat GTGTC CCCACTCTTT GGAACTATCT

Rat cDNA AGCTGCCAAC TTGGGGCCCG AGCAG

Mouse AACTGCCAAC TTGGAGCCAG AGCAGGTGTC CCCACTCTTT GGAACTATCT

Dog GGCTGCCCAC CTGGAGCCAG AGCAGGTGTC CCCGCTCTTT GGGACCATCT

Treeshrew GTGTC CCCACTCTTT GGAACCGTCT

Mouse lemur GTGTC CCCACTCTTT GGAACCATCT

Bushbaby GGCTTCCCAC CTGGAGCCAG ACCAG

Macaque AGCTGCCCAC CTGGGGCCAG AGCAG

Orangutan GGCTGCCTAC CTGGAGCCAG AGCAG

Sumatran orangutan GGCTGCCTAC CTGGAGCCAG AGCAG

Chimpanzee GGCTGCCCAC CTGGAGCCAG AGCAG

Human GGCTGCCCAC CTTGAGCCAG AGCAG

ex3 <-||-> ex4

1182 1192 1202 1212 1222

| | | | |

D A V I L L A H A L N H S E T H

Rat ACGATGCCGT CATCCTGTTG GCCCATGCCC TGAACCACTC TGAGGCCCAT

Mouse ATGATGCCGT CATCCTGTTG GCCCATGCCC TGAACCGATC TGAGACCCAT

Dog ATGATGCTGT TGTCCTGCTG GCCCACGCCC TGAACCGCTC TGAGAGCCAT

Treeshrew ATGATGCTGT TGTCCTGTTG GCCCATGCAT TGAACCACTC TGAGAGCCAT

Mouse lemur ATGACGCTGT TGTCCTGCTG GCCCATGCCC TGAACCGCTC TGAGAGCTAT

1232 1242 1252 1262 1272

| | | | |

G T G L S G A H L G N H I R A L D

Rat GGAACGGGGC TCTCAGGGGC TCACTTAGGG AACCATATAA GAGCTCTTGA

Mouse GGAGCGGGGC TCTCAGGGGC TCACTTAGGG GACCACGTAA GAGCTCTTGA

Dog GGTGCAGGAC TCTCAGGGGC CCGTTTGGGG GACCATACTG GGGCTCTGAA

Treeshrew GGGGATGGGC TCTCAGGGGC CCACCTAGGG GACCACATGG GCACCCTGGA

Mouse lemur GGGGCAGGGC TCTCGGGGGC CCACTTAGGA GATCACACGC GGGCTCTGGA

1282 1292 1302 1312 1322

| | | | |

V A G F S Q R I R I D G K G R R L

Rat TGTGGCTGGT TTTAGCCAGA GAATCCGGAT AGATGGGAAA GGCAGAAGGC

Mouse TGTGGCTGGT TTTAGCCAGA GAATCCGAAC AGATGGGAAA GGCAGAAGGC

Dog TGTGGCTGGC TTTAGCCAGA GGATCCGGAC AGATGAGAAG GGCAGGAGGC

Treeshrew TGTAGCTGGC TATAGCCAGA GGATCCGGAC GGATGTGAAG GGCCGGAGGC

Mouse lemur TGTGGCCGGC TTTAGCCAGA GGATCCGGAC AGACGAGAAA GGCAGGAGGC

1332 1342 1352 1362 1372

| | | | |

P Q Y V I L D T N G E G S Q L V

Rat TACCCCAATA CGTCATCCTG GACACAAATG GTGAAGGAAG CCAGTTGGTT

Mouse TAGCCCAATA TGTCATCCTG GACACAGATG GTGAAGGAAG CCAGTTGGTT

Dog TGGCTCAGTA TGTCATTTTG GACACAGATG GTCGGGGAAG CCAGCTGGTC

Treeshrew TGGCCCAGTA TGTCATTCTG GACACGAAGG GTCAAGGAAG CCAGCTGGTC

Mouse lemur TGGCCCAGTA CATCATCCTG GACACAGATG GCCAAGGAAG CCAGCTGGTC

1382 1392 1402 1412 1422

| | | | |

P T H I L D V S T Q Q V Q P L G T

Rat CCCACCCACA TCCTGGACGT GAGCACACAG CAGGTGCAGC CCCTGGGAAC

Mouse CCTACCCACA TTCTGGACAC AAGCACATGG CAGGTGCAGC CTCTGGGAAA

Dog CCCACTCACA TTCTGGACAC AGGCACATGG CAAGTGCAGC CCCTGGACAG

Treeshrew CCCACACACA TCTTGGAC

Mouse lemur CCCACCCACA CCCTGGACAC AGGCACCTGG CAAGTGCAGC CGCTGGGCAG

1432 1442 1452 1462 1472

| | | | |

A V H F P G G S P P A H D A S C W

Rat GGCGGTACAC TTCCCTGGAG GGAGCCCTCC AGCCCGTGAT GCCAGCTGCT

Mouse GCCTATACAC TTTCCTGGGG GGAGCCCTCC AGCCCATGAC GCCAGCTGCT

Dog GGCCATACAC TTTCCAGGAG GGGCCCCTCC GGCACGTGAC TCCAGCTGCT

Mouse lemur GGCCATACAC TTTCCAGGAG GAGCCCCTCC GGCCCATGAC TCCAGCTGCT

1482 1492 1502 1512 1522

| | | | |

F D P N T L C I R G V Q P L G S

Rat GGTTCGACCC CAACACACTA TGCATACGAG GTGTGCAGCC CCTGGGCAGC

Mouse GGTTCGACCC CAACACACTA TGCATCAGAG GTGTGCAGCC CCTGGGCAGC

Dog GGTTTGACCC CAAAATGCTA TGCGTGAGAG ATGTGCAGCC CCCGGGCAGC

Mouse lemur GGTTTGACCC AGATACACTG TGCATGAGAG ATGCGCGGCC CCTGGGCAGC

ex4 <-| |-> ex5

1532 1542 1552 1562 1572

| | | | |

L L T L T I T C V L A L V G G F L

Rat CTCCTGACTC TGACGATAAC CTGTGTCCTG GCTCTGGTTG GTGGATTCCT

Mouse CTCCTGACTC TGACGATAGC CTGTGTCCTG GCACTGGTTG GTGGATTCCT

Dog CTCATCGCTT TTGCCCTGGC CTGTATCCTG GTACTGGCCG GCGGGGCCCT

Mouse lemur CTGCCCGCTC TGGCGCTGGC CTGCCTCCTG GCGCTGGCCG GCGGGGCCCT

1582 1592 1602 1612 1622

| | | | |

A Y F I R L G L Q Q L R L L R G P

Rat TGCTTACTTT ATCAGGTTAG GCCTCCAGCA GCTGCGGCTG CTGCGGGGCC

Mouse TGCTTACTTC ATCAGGTTAG GCCTCCAGCA GCTGCGGCTG TTGCGGGGCC

Dog CACTTGCCTC ATCAGATTGG GCATCCAACA GCTGCGGCTG GTGCGGGGCC

Treeshrew GTTGG GTGTCCAGGA GGCGCAGTTG GTGCGGGGCC

Mouse lemur CGCTTGGCTC CTCAGATTGT GCATCCAGCG GCTGCGGCTA GTGCGGGGTC

Bushbaby ATTGG GTATCCGGCA GCTGCGGCTG GTACGGGGCC

Tarsier ATTGG GCATCCAGAA GGTGCAGCTG GTGTGGGGGC

Macaque ATTGG GCATCCAGCA GCTGCAGCTG GTGTGGG--C

Orangutan TTTGG GCATCCAGCA GCTGCAGCTG GTGTGGGGCC

Chimpanzee g gcatccacca gctgcagctg gTGTGGGGCC

Human TTTGG GCATCCACCA GCTGCAGCTG GTGTGGGGCC

ex5 <-||-> ex6 6A

1632 1642 1652 1662 1672

| | | | |

H R I L L T P Q E L T F L Q R T

Rat CCCATCGGAT CCTGCTGACA CCTCAAGAGC TCACCTTCCT CCAGCGAACC

Mouse CCCATCGGAT CCTGCTGACA TCTCAGGAGC TCACCTTCCT CCAGCGGACC

Dog CCCACCGGAT CCTGCTGACA GCCCAGGAGC TCACCTTCAT CCATCGGCCC

Treeshrew CCCCCGGATT CCTGCTCCCA GCCCCAGGAA TCACCTTCCT CCATCAGCCC

Mouse lemur GCCACCGGAT CCTGCTGACC GCCCAGGAGC TCACCTTCGT CCATCGGCCC

Bushbaby CTCACCGGAT CCTGCTGACA GCCCAAGAGC TCACCTTTAT CCAT---ACC

Tarsier CCCACCGGAT CCTGCTGACA GCCCAGGAGC TCGCCTTCTT CCATCTGTCC

Macaque GCCACCAGAT CCTGCTGACA GCCCAGGAGC TCACCTTCAT CCATTCGCCC

Orangutan CCCACGGGAT CCTGCTGACA GCCCGGGAGC TCACCTTCGT CCATCCACCC

Chimpanzee CCCACCGGAT CCTGCTGACA GCCCAGGAGC TCACTTTCAT CCATCCACCC

Human CCCACCGGAT CCTGCTGACA GCCCAGGAGC TCACCTTCAT CCATCCACCC

1682 1692 1702 1712 1722

| | | | |

P S R R R P H V D S G S E S R S V

Rat CCCAGCCGGC GGAGGCCACA TGTGGACAGT GGCAGCGAGT CAAGAAGTGT

Mouse CCCAGCCGGA GGAGGCCACA TGTGGACAGC GGCAGTGAGT CAAGAAGTGT

Dog CCGAGCAGAC GGCGGCTGCA CATGGACAGT GTGAGCGATT CAAGGAGTGC

Treeshrew CCCAGTAGGC GGAGGCTTCA CGTGGATAGT GCAAGTGAGT CCAGAAGTGT

Mouse lemur CTGAGCAGAC GGAGACTGCG TGTGGACAGT G------AGT CAAGAAGTGT

Bushbaby CCAAGCAGAT GGAGGCTGCA TATGGACAGT G------AGT CAAGAAGTGC

Tarsier CCAAGCAGAT GGAGACTGCA TGTGGACAGT A------AGT CGAGAAGTGT

Macaque CTGAACAGAT GGAGGCTGCA TGTGAATGAT G------AAT GGGGAATTGT

Orangutan CTGAACAGAT GGAGGCTGCA TGTGGATGCT G------AAT GGAGAAGGGT

Sumatran orangutan AGGCTGCA TGTGGATGCT G------AAT GGAGAAGGGT

Chimpanzee CTGAACAGAC AGAGGCTGCA TGTGGATGGT G------AAT GGAGAAGTGT

Human CTGAACAGAC AGAGGCTGCA TGTGGATGGT G------AAT GGAGAAGTGT

ex6 <-||-> ex7

1732 1742 1752 1762 1772

| | | | |

V D G G S P Q S V I Q G S T R S V

Rat GGTAGATGGT GGGAGTCCAC AGTCAGTGAT CCAGGGGTCA ACAAGGAGCG

Mouse GGTGGATGGT GGAAGTCCAC GGTCGGTGAC CCAGGGGTCA GCAAGGAGCC

Dog GGTGGAAGGT GGGAGCCTGA GGTCGGTGGC CCAGGGGTCA GCAAGGAGCC

Treeshrew GGGCGATGTT GGGAGCCTGA GGTCAGGGGC CCAGGGCTCA GCCAGGAGCC

Mouse lemur GGTGGATGGT GGGAGCCTGA AGTCGGTGGC CCAGGGCTCA GCAAGGAGCC

Bushbaby AGCAGATGGT GGGAGCCTGA GATCCATGGC TCAAGGGTCA GCA

Tarsier GGTGGATGGC AGAAGCCTGA TGTCAGTGGC CCAGGGGTCA GCAAAGAGCC

Macaque GGCAGATGGT GGGAGCCTGA GATCAGTGGC CCGGGGATCA GCAAAGAGTC

Orangutan GGCGGATGGT GGGAGCCTGA GGTCAGTGGC CCAGCTGTCA GCAAAGAGTC

Sumatran orangutan GGCGGATGGT GGGAGCCTGA GGTCAGTGGC CCAGCTGTCA GCAAAGAGTC

Chimpanzee GGCGGATGGT GGGAGCCTGA GGTCAGTGGC CCAGGGGTCA GCAAAGAGTC

Human GGCGGATGGT GGGAGCCTGA GGTCAGTGGC CCAGGGGTCA GCAAAGAGTC

1782 1792 1802 1812 1822

| | | | |

P A F L E H T N V A L Y Q G E W

Rat TACCAGCCTT CCTGGAGCAC ACCAACGTGG CCCTGTACCA GGGAGAGTGG

Mouse TACCAGCCTT CCTGGAGCAC ACCAACGTGG CCCTGTACCA GGGAGAGTGG

Dog TGCTAGCTCC CCAGGAACCC ACCAATGTGG CCCTGTACCA GGGGGAGTGG

Treeshrew TGCCAGCCCC CCAGGAGCCC ACCAGCGTGG CCCTATACCA GGGAGACTGG

Mouse lemur CGCCAGCCCC CCAGGAGCCC ACCAACGTGG CCCTGTACCA GGGAGACTGG

Tarsier TGCCAGGTTC CCAGAAACCA ATCTGCATGG CCCTGTATCA G

Macaque TGCCAGCC-C CCAGGAACCC ACCAGTGTGG CCGTGTACCA AGGAGACTGG

Orangutan TGCCAGCCCC CTGGGAACCC ACCAGTGTGG CCCTGTACCA AGGAGACTGG

Sumatran orangutan TGCCAGCCCC CTGGGAACCC ACCAGTGTGG CCCTGTACCA AGGAGACTGG

Chimpanzee TGCCAGCCCC CCAGGAACCC ACCAATGTGG CCCTGTACCA AGGATACTGG

Human TGCCAGCCCC CCGGGAACCC ACCAATGTGG CCCTGTACCA AGGAGACCGG

7A ex7 <-||-> ex8

1832 1842 1852 1862 1872

| | | | |

V W L K K F E A G T A P D L R P S

Rat GTGTGGCTGA AGAAGTTTGA GGCAGGCACG GCTCCTGATC TGCGGCCCAG

Mouse GTATGGCTGA AGAAGTTTGA AGCAGGCGTG GCTCCTGATC TTCGGCCAAG

Dog GTGTGGCTGA AGAGGTTTGA AGCGGGGACT GCCCCGGAGC TGCGGCCCAG

Treeshrew GTGTGGCTGA AGAAGTTTGA AGCAGGCACA GCCCCCGAGC TGCGTCCGGG

Mouse lemur GTGTGGCTGA AGAGGTTCGA AGTGGGCCTG CCACCCGAGC TGCGCCCGAG

Macaque GTGTGGCTGA AGAAGTGTGA AGTGGGCTAA GACCTGGAAC TGCGCCTGAG

Orangutan GTGTGGCTGA AGGAGTGTGA AATGGGCTCA GACCTTGAGC TGCACCCAAG

Sumatran orangutan GTGTGGCTGA AGGAGT

Chimpanzee GTGTGGCTGA AGAAGTGTGA AGTGGGCTCA GACCCTGAGC TGCGCCCAAG

Human GTGTGGCTGA AGAAGTGTGA AGTGGGCTCA GACCCTGAGC TGCGCCCAAG

8A

1882 1892 1902 1912 1922

| | | | |

S L S L L R K M R E M R H E N V T

Rat CAGCCTCAGC CTCCTGAGAA AGATGCGGGA GATGCGGCAT GAGAATGTCA

Mouse TAGCCTTAGC TTCCTGAGAA AGTTGCGGGA GATGCGGCAT GAGAATGTTA

Dog CTGCCTCAGC CTCCTGAGAA AGATACGGGA GCTGAGGCAT GAGAACGTTG

Treeshrew TTGCCTCAGC CTCCTGAGAA AGATGCGGGA ACTTCGGCAT GAGAACGTCA

Mouse lemur CTGCCTCCGC CTCCTGAGAA AGATGCGGGA GCTTCAGCAT GAGAACGTTG

Bushbaby GTCA

Common marmoset ATGTGGGA GCTGCGCCAT GAGAATGTTG

Drill AACGTCG

Macaque CTGCCTCAGC CTCCTGAGGA AGATGTGGGA GCTGCAGTGT GAGAACGTCA

Orangutan CTGTCTCAGC CTCCTGAGGA AGATGTGGGA GCCACAGTGT GAGAACGTCG

Sumatran orangutan ATGTGGGA GCCGCAGTGT GAGAACGTCA

Chimpanzee CTGCCTCAGC CTCCCGAGGA AGATGTGGGA GCTGCAGTGT GAGAACGTTG

Bonobo AACGTCG

Human CTGCCTCAGC CTCCCGAGGA AGATGCGGGA GCTGCGGTGT GAGAACGTCG

ex8 <-||-> ex9

1932 1942 1952 1962 1972

| | | | |

A F L G L F V G P E V S A M V L

Rat CCGCCTTCCT GGGTCTCTTT GTGGGCCCTG AGGTTAGTGC GATGGTGCTG

Mouse CTGCCTTCCT GGGCCTCTTT GTGGGCCCTG GGGTCAGTGC AATGGTGCTG

Dog CTGCCTGCCT GGGAGTCTTT GTGGCCCCTG GGGTCAGCGC GCTCGTGCTG

Treeshrew CCACCTGCCT GGGTTTTTTT GTGGCCCCAG GGATCAGTGC TCTGGTGCTG

Mouse lemur CCACCTGCCT GGGCGTTTTC GTGGCCCCTG GCGCCAGCGC GCTGGTACTG

Bushbaby CTGCCTGCCT GGGCTTTTTC ACGGCCCCTG GGGTCAGTGC TCTGGTTCTG

Common marmoset CCACCTGCCT AGGCTTTTTC GTGAAC-CTG GGGTCAGTGC TCTGTTTCTG

Drill CCAC-----T GGGCTTTTTC GTGACCCCTG GGGTGAGTGC TCTGGTTCTG

Macaque CCAC-----T GGGCTTTTTC GTGACCCCTG GGGTCAGTGC TCTGGTTCTG

Siamang GCTTTTTC ATGACCTCTG GGG------- ----------

Orangutan CCACCTGCCT GGGCTTTTTT GTGACTCCTG GGG------- ----------

Sumatran orangutan CCACCTGCCT GGGCTTTTTT GTGACTCCTG GGG------- ----------

Gorilla ACCTGCCT GGGCATTTTC GTGATCTCTG GGG------- ----------

Chimpanzee CCACCTGCCT GGGCATTTTC GTGATCTCTG GGG------- ----------

Bonobo CCACCTGCCT GGGCATTTTC GTGATCTCTG GGG------- ----------

Human CCACCTGCCT GGGCATTTTC GTGATCTCTG GGG------- ----------

9A 9B 9C

1982 1992 2002 2011 2021

| | | | |

E H C A R G S L E D L L R N E D L

Rat GAGCACTGTG CCCGTGGCAG CCTGGAG-GA CCTGCTACGG AATGAGGACC

Mouse GAGCACTGTG CCCGTGGCAG CCTGGAG-GA CCTGCTGCAG AATGAGAACC

Dog GAGCACTGTG CCCGGGGCAG CCTGGAG-GA CCTGCTGCGG AATGAAGCTC

Treeshrew GAGCACTGTG CCCGGGGCAG CCTGGAG-GA CTTGCTGCGG AATGAGGCCC

Mouse lemur GAGCACTGCC CCCGGGGCAG CCTGGAG-GA CCTGCTACGG AACGAGGCCC

Bushbaby GAGCACTGTC CCCGGGGCAG CCTGGAG-AA CCTGCTACGG AATGAGGCCC

Common marmoset GAGCACTGCC CACAAGGCAG CCTGGAACGA AGTCCTGTGG AATGAAGTCC

Drill GAG------- ---------- --------AA CCTGCTGCGG AACGAGGCTC

Macaque GAG------- ---------- --------AA CCTGCTGCGG AACGAGGCTC

Siamang ---------- ---------- --------AA CCTGCTGCGG AAAGAGGTCC

Orangutan ---------- ---------- --------AA CCTGCTGCGG AATGAGGTCC

Sumatran orangutan ---------- ---------- --------AA CCTGCTGCGG AATGAGGTCC

Gorilla ---------- ---------- --------AA CCTGCTGCGG AAAGAGGTCC

Chimpanzee ---------- ---------- --------AA CCTGCTGCGG AAAGAGGTCC

Bonobo ---------- ---------- ---------A CCTGCTGCGG AAAGAGGTCC

Human ---------- ---------- --------AA CCTGCTTCGG AAAGAGGTCC

9D9E

2031 2041 2051 2061 2071

| | | | |

R L D W T F K A S L L L D L I R

Rat TGAGGCTAGA CTGGACCTTC AAGGCCTCCT TACTGTTGGA TCTGATCCGA

Mouse TGAGGCTAGA CTGGACCTTC AAGGCCTCCT TACTGTTGGA TCTGATCCGA

Dog TGCGGCTGGA TTGGACCTTC AAGGCCTCTC TGTTGCTGGA TTTGATCCGT

Treeshrew TGAGGCTAGA CTGGACCTTC AAGGCCTCAC TGCTGCTGGA CTTGATCCGA

Mouse lemur TGCCGCTAGA CTGGACCTTC AAGGCCTCCC TGCTGCTGGA TTTGATCCGC

Bushbaby TGCGGCTAGA CTGGACCTTC AAGGCCTCCC TGTTGCTGGA TTTGATCCGT

Common marmoset TGCACCTAGA CTGGACCTTC AAGGCCTCCC TGA-ACTGGA TTTGATCCAT

Drill TGCACCTAGA CTGGACATTC AAGGCCTCCC TGCTGCTGGA TTTGATCTGC

Macaque TGCACCTAGA CTGGACCTTC AAGGCCTCCC TGCTGCTGGA TTTGATCTGC

Siamang TGCACCTAGA CTGGACCTTC AAGGCCCCCC TGCTGCTGGA TTTGATCTGC

Orangutan TGCACCTAGA CTGGACCTTC AAGGCCTCCC TGCTGCTGGA TTTGATCTGC

Sumatran orangutan TGCACCTAGA CTGGACCTTC AAGGCCTCCC TGCTGCTGGA TTTGATCTGC

Gorilla TGCACCTAGA CTGGACCTTC AAGGCCTCCC

Chimpanzee TGCACCTAGA CTGGACCTTC AAGGCCTCCC TGCTGCTGGA TTTGATCTGC

Bonobo TGCACCTAGA CTGGACCTTC AAGGCCTCTC TGCTGCTGGA TTTGATCTGC

Human TGCACCTAGA CTGGACCTTC AAGGCCTCCC TGCTGCTGGA TTTGATCTGC

9F ex9 <-|

2081 2091 2101 2111 2121

| | | | |

G L R Y L H H R H F P H G R L K S

Rat GGTTTGCGGT ATCTGCACCA TCGACATTTC CCCCATGGGC GCCTCAAGTC

Mouse GGTTTGCGGT ATCTGCACCA TCGGCGCTTC CCCCACGGGC GCCTCAAGTC

Dog GGCATGAGGT ATCTGCACCA TCGGCATTTC CCTCACGGCC GCCTCAAGTC

Treeshrew GGCATGAGGT ATCTGCACCA TCGACATTTC CCTCATGGCC GCCTCAAGTC

Mouse lemur GGCATGCGGT ATCTGCACCA TCGAGGTTTC CCCCACGGCC GCCTCAAGTC

Bushbaby GGCATGCGGT ACCTGCACTG TCGATGTTTC CCTCATGGTC GCCTCAAGTC

Tarsier GGCATGCAGT ATCTTTACCA TCGACATTTC CCTCACGGCC ACCTCAAGTC

Titi TTCTGGCT GCCTCAAGTC

Owl monkey CTGGCT GCCTCAAGTC

Red-backed squ monkey TTCTGGCT GCCTCAAGTC

Common squirrel m CTGGCT GCCTCAAGTC

Common marmoset GGCATGAGAT ATCTGTACCA CCGACATTTC CCTCATGGCC GCCTCAAGTC

Pygmy marmoset TTCTGGCT GCCTCAAGTC

Drill TCCTGGCT GCCTCAAGTC

Macaque GGCATGAGGT ATCTGCACCA CTGATGTTTC CCTCCTGGCT GCCTCAAGTC

Siamang TCCTGGCT GCCTCAAGTT

Orangutan GGCGTGAGGT ATCTGCATCA CCAACATTTC CCTCTTGGCT GCCTCAAGTT

Sumatran orangutan GGCGTGAGGT ATCTGCATCA CCAACATTTC CCTCTTGGCT GCCTCAAGTT

Gorilla TCCTGGCT GCCTCAAGTT

Chimpanzee GGCGTGAGGT ATCTGCACCA CCACCATTTC CCTCCTGGCT GCCTCAAGTT

Bonobo TCCTGGCT GCCTCAAGTT

Human GGCATGAGGT ATCTGCACCA CCACCATTTC CCTCCTGGCT GCCTCAAGTT

|-> ex10 10A

2131 2141 2151 2161 2171

| | | | |

R N C V V D T R F V L K I T D H G

Rat CAGGAACTGT GTGGTGGACA CTCGCTTTGT GCTCAAGATC ACTGACCATG

Mouse CAGGAACTGT GTGGTGGACA CTCGCTTTGT GCTCAAGATC ACTGATCATG

Dog CCGAAACTGT GTGGTGGATG GACGCTTTGT TCTCAAGGTC ACTGACCATG

Treeshrew CCGGAACTGC GTAGTGGATG GACGCTTCGT GCTGAAGGTC ACTGACCATG

Mouse lemur CCGAAACTGC GTGGTGGACG GTCGCTTTGT GCTCAAAGTC ACTGACCACG

Bushbaby CCGAAACTGT GTCGTGGACA GCCACTTTGT GCTCAAAGTC ACCGACCATG

Tarsier CCGAAACTGT GTGGTGGACT GTCGCTTTGT GCTCAAGGTC ACTGACCACG

Titi CTGAAACTGT GTGGTGGACA GATGCTTTGT GCTCAAGGCC A-TGACCACG

Owl monkey CCGAAACTGT GTGGTGGACA GATGCTTTGT GCTCAAGACC ACTGACCATG

Red-backed squ monkey CCGAAACTGT GTGGTGGACA GATGCTTTGT GCTCAAGACC ACTGACCATG

Common squirrel m CCGAAACTGT GTGGTGGACA GATGCTTTGT GCTCAAGACC ACTGACCATG

Common marmoset CCAAAACTGT GTGGTGGACA GACGCTTTGT GCTCAAGACC ACTGACCATG

Pygmy marmoset CCAAAACTGT GTGGTGGACA GACGCTTTGT GCTCAAGACC ACTGACCATG

Drill CCAAAACTGT GTGGTGGAMA GACACTTTGT GCTCAAGGTC ACTGACCATG

Macaque CCAAAACTGT GTGGTGGACA GACACTTTGT GCTCAAGGTC ACTGACCATG

Siamang CCAAAACTG- ---GTGGACA GACGCTTTGT GCTCAAGGTC ACTGACCATG

Orangutan CGAAAACTGT G--GTAGACA GACGCTTTGT GCTCAAGGTC ACTGACCATG

Sumatran orangutan CGAAAACTGT G--GTAGACA GACGCTTTGT GCTCAAGGTC ACTGACCATG

Gorilla CCAAAACTGT GTGGTGGACA GATGCTTTGT GCTCAGGGTC ACTGACCATG

Chimpanzee CCAAAACTGT GTGGTGGACA GATGCTTTGT GCTCAAGGTC ACTGACCATG

Bonobo CCAAAACTGT GTGGTGGACA GATGCTTTGT GCTCAAGGTC ACTGACCATG

Human CCAAAACTGT GTGGTGGACA GATGCTTTGT GCTCAAGGTC ACTGACCATG

10B 10C 10D 10E

2181 2191 2201 2211 2221

| | | | |

Y A E F L E S H C S F R P Q P A

Rat GTTATGCAGA GTTCCTGGAG TCTCACTGCT CTTTCAGGCC CCAGCCGGCC

Mouse GCTATGCAGA GTTCCTGGAG TCTCACTGTT CTTCCAGGCC CCAGCCAGCC

Dog GTTATGCGGA ACTCCTGGAT GCTCAGCGGG CTCCCCGCCC CCGGCCAGCC

Treeshrew GCTACGCAGA GCTCCTGGAC ACCCAGCGTG CTGCCTGCCC CCGGCCAGCC

Mouse lemur GTTATGCAGC ACTCCTGGAC GCTCAGCGGG CTCCACGACC CCGGCCAGCC

Bushbaby GTTATGCAGC GCTCCTGGAT GCTCAGCAGG CTCCCCAACC CCAGCCAGTC

Tarsier GCTACGCAGC ACTCCTGGAA GCTCAGCGGG CTCCCTGACC CTGGTCAGCC

Titi GTTATGCAAA GCTCATGGAC ACTCCGCAGG CTCCCCAACC CTGGCCAGCC

Owl monkey GTTATGCAGA GCTCCTGGAC ACTCAGCAGG CTCCCCAACC CTGGCCAG-C

Red-backed squ monkey GTTATGCAGA GCTACTGGAC ACTCAGCGGG –TCCCCAGAC CTGGCCAG-C

Common squirrel m GTTATGCAGA GCTACTGGAC ACTCAGCGGG CTCCCCAGAC CTGGCCAG-C

Common marmoset GTTATGCAGA GCTCCTGGAC ACTCAGCAGG CTCCCCAACC CTGGCCAG-C

Pygmy marmoset GTTATGCAGA GCTCCTGGAC ACTCAGCAGG CTTCCCAACC CTGGCCAG-C

Drill GTTATGCAGA GCCCCTGGAC ACTCAGCAGG CTCCCCAACC CTGGCCAGCC

Macaque GTTATGCAGA GCCCCTGGAC ACTCAGTAGG CTCCCCAACC CTGGCCAGCC

Siamang GTTATGCAGA GCCCCCGGAC ACTTAGCAGG CTCCCCAACC CTGGCCAGCC

Orangutan GTTATGCAGA GCCCCTGGAC ACTCAGCAGG CTCCCCAACC CTGGCCAGCC

Sumatran orangutan GTTATGCAGA GCCCCTGGAC ACTCAGCAGG CTCCCCAACC CTGGCCAGCC

Gorilla GTTATGCAGA GCCCCTGGAC ACTTAGCAGG ATCCCCAACC CTGGCCAGCC

Chimpanzee GTTATGCAGA GCCCCTGGAC ACTTAGCAGG ATCCCCAACC CTGGCCAGCC

Bonobo GTTATGCAGA GCCCCTGGAC ACTTAGCAGG ATCCCCAACC CTGGCCAGCC

Human GTTATGCAGA GCCCCTGGAC ACTTAGCAGG ATCCCCAACC CTGGCCAGCC

10F 10G 10H 10I 10J

2231 2241 2251 2261 2271

| | | | |

P E E L L W T A P E L L R G P R G

Rat CCAGAAGAGC TTCTGTGGAC GGCTCCTGAG CTGTTGCGGG GGCCTCGGAG

Mouse CCAGAAGAGC TTCTGTGGAC GGCTCCTGAG CTGCTGCGGG GGCCT-----

Dog CCAGAAGAGC TGCTGTGGAC GGCTCCGGAG CTGCTNNNNN NNNNN-----

Treeshrew CCAGAAGAAC TGCTATGGAC GGCTCCTGAA CTGCTCCGAG GCCCT-----

Mouse lemur CCAGAAGAGC TGCTGTGGAC GGCTCCTGAG CTGCTGCGGG AGCCT-----

Bushbaby CCAGAAGAGC TTCTGTGGAC GGCTCCTGAG CTGCTGCGAT TGCCT-----

Tarsier CTGGAAGAGC TGCTGTGAAC GGCTCCTGAG TTGCTGTGGA GGCCT-----

Titi CTGGAAGAGC TGCTGTGGAC AGCTCCTGAG CTGCCACGGG GGCCT-----

Owl monkey CTGGAAGAGC TGCTGTGGAC AGCTCCTGAG CTGCCGCGAG GGCCT-----

Red-backed squ monkey CTGGAAGAGC TGCTGTGGAC AGCTCCTGAG CTGCCGCGAG GGCCT-----

Common squirrel m CTGGAAGAGC TGCTGTGGAC AGCTCCTGAG CTGCCGCGAG GGCCT-----

Common marmoset CTGGAAGAGC TGCTGTGAC- ---------- ---------- GGTCT-----

Pygmy marmoset CTGGAAGAGC TGCTGTGAG- ---------- ---------- GGTCT-----

Drill CCCGAATAGC TGCTATGGGC AGCTCCTGAG CTGCTGTCGA GGCCT-----

Macaque CCCGAATAGC TGCTATGGGC AGCTCCTGAG CTGCTGCCGA GGCCT-----

Siamang ATGGAAGAGC TGCT------ ---------- -----GCCGA GGCCT-----

Orangutan CCGGAAGAGC TGCTATGGAC AGCTCCTGAG CTGCTGCCGG GGCCT-----

Sumatran orangutan CCGGAAGAGC TGCTATGGAC AGCTCCTGAG CTGCTGCCGG GGCCT-----

Gorilla CTGGAAGANC TGCTATGGAC AGCTCCTGAA C

Chimpanzee CCGGAAGAGC TGCTATGGAC AGCTCCTGAG CTGCTGCTGG GGCCT-----

Bonobo CCGGAAGAGC TGCTATGGAC AGCTCCTGAG CTGCTGCCGG GGCCT-----

Human CCAGAAGAGC TGCTATGGAC AGCTCCTGAG CTGCTGCCGG GGCCT-----

ex10 <-||-> ex11

10K 11A

2281 2291 2301 2311 2321

| | | | |

P W G P G K A T F K G D V F S L G

Rat GCCTTGGGGG CCTGGGAAGG CCACCTTCAA AGGTGATGTT TTCAGCCTGG

Mouse ---------- ---GGGAAGG CCACCTTCAA AGGTGATGTT TTCAGCTTGG

Dog ----NNNNNN NNNNNNNNNN NNACCCTCAA AGCAGACATC TTCAGCATCG

Treeshrew ----GGGGCC CCCGGGCGGG GCACCTTCAA AGGAGACATC TTCAGCATCG

Mouse lemur ----GCAGGG CCCGGGCCGG GCACCTTCAA GGGGGACATC TTCAGTATTG

Bushbaby ----GCAGGC CCAGGGAGGG GCCCACTCAA AGGATACGTC TTCAGCATTG

Tarsier ----GGGCTC TCCGGGCAGG GCACCCTCAA AGGGGACGTC TTCAGCATCG

Titi ----AGGTGC TCTGGGCAGG GCACGCTCAC AGGGGACATC TTCAGCATTG

Owl monkey ----GGGCGC CCCGGGCGGG GCACCCTCAC AGGGGACATC TTCAGCACTG

Red-backed squ monkey ----GGGTGC CCC----AGG GCACCCTCAC AGGGGACATC TTCAGCACTG

Common squirrel m ----GGGTGC CCC----AGG GCACCCTCAC AGGGGACATC TTCAGCACTG

Common marmoset ----GGGCGC CCTGGGCGGG GCACCCTCAC AGGGGACATC TTCAGCACTG

Pygmy marmoset ----GGGGGC CCTGGGCGGG GCACCCTCAC AGGGGACATC TTCGGCACTG

Drill ----GGGTGC CCTGGGTGAG GCACCCTCAC AGGGGACATC TTCAGCACTG

Macaque ----GGGTGC CCTGGGTGGG GCACCCTCAC AGGGGACATC TTCAGCACTG

Siamang ----GGGTGC CCTGGGCGGG GCACCCTCAC AGGTGACATC TTCAGCACCG

Orangutan ----GGGCGC CCTGGGCGGA GCACCCTCAC AGGGGACATC TTCAGC--TG

Sumatran orangutan ----GGGCGC CCTGGGCGGA GCACCCTCAC AGGGGACATC TTCAGC--TG

Chimpanzee ----GGGCGC CCTGGGTGGC ACACCCTCAC AGGGGACATC TTCAGCACTG

Bonobo ----GGGCGC CCTGGGTGGC ACACCCTCAC AGGGGACATC TTCAGCACTG

Human ----GGGCGC CCTGGGCGGC GCACCCTCAC AGGGGACATC TTCAGCACTG

11B 11C 11D

2331 2341 2351 2361 2371

| | | | |

I I L Q E V L T R D P P Y C S W

Rat GCATCATCCT GCAGGAGGTA CTGACCCGAG ACCCACCCTA CTGCTCCTGG

Mouse CCATCATCCT GCAGGAGGTA CTGACCCGAG ACCCACCCTA CTGCTCCTGG

Dog GCATCGTCCT ACAGGAGGTG CTGACTCGAG GCCCACCCTA CAGCTCCTCG

Treeshrew GCATCATTCT GCAGGAGGTG CTGACTCGAG GCCCTCCCTA CTGCTCCTCG

Mouse lemur GCGTCATCCT GCAGGAGGTG CTGACGCGAG GCCCGCCCTA CTGCTCCTTG

Bushbaby GTATCATCCT GCAGAGGGAG CTGAGTCGAG ATGCGCCCTA CTGCTCCTCG

Tarsier GCATCATCCT GCAGGAGGTG CTGACCCGAG GTCCGC-CTA CTGCTCCTCA

Titi G

Owl monkey G

Red-backed squ monkey G

Common squirrel m G

Common marmoset GCATTATCCT GCAGGAGGTG CTAACTCAGG GTCCACCCTA CTGCTCCCTG

Pygmy marmoset G

Drill CCATCATCCT GCAGGANGTG CTGCCTCGGG ACCCACC

Macaque CCATCATCCT GCAGGAGGTG CTGCCTCGGG ACCCACCCTA CTGCTCCTCG

Siamang GCATCATCCT GCAGGAGGTG CTGACTCGGG GCCCACC

Orangutan GCATCATCCT GCAGGAGGTG CTGACTCGGG GCCCACCCTA CCGCTCCTCA

Sumatran orangutan GCATCATCCT GCAGGAGGTG CTGACTCGGG GCCCACCCTA CCGCTCCTCA

Chimpanzee GCATCATCCT GCAGGAGGTG CTGACTTGGG GCCCACTCTA CTGCTCCTCG

Bonobo GCATCATCCT GCAGGANGTG CTGACTTGGG GCCCACT

Human GCATCATCCT GCAGGAGGTG CTGACTCG-G GCCCACTCTA CTGCTCCTCG

11E 11F

2381 2391 2401 2411 2421

| | | | |

G L S A E E I I R K V A S P P P L

Rat GGACTCTCAG CAGAAGAGAT CATCCGGAAG GTGGCATCTC CCCCTCCTCT

Mouse GGACTCTCAG CAGAAGAGAT CATCCGGAAG GTGGCATCTC CCCCTCCTCT

Dog GGGCTCTCCG CAGAAGAAAT CATCAGGAAG GTGGTATCAC CCCCTCCCTT

Treeshrew GCACTCTCAG CAGAAGAAAT CATCAGGAAG GTAATGTCCC CACCCCCGCT

Mouse lemur GGACTCTCAG CAGAAGAAAT CATCAGGAAG GTGGCATCTC CCTCTCCTTT

Bushbaby GAACTGACAG CAGAGGAAAT CATCAGGAAG GTGGCATCTT TCCCTCCCTT

Tarsier GGACTGTCAG CGGAAGAAAC CATCAGGAAG GTGGCGACTC TACTTCCCCT

Common marmoset GGACTCCCAG TGAAAG

Drill CCCTCCTCT

Macaque GGATTCCCAG TGGAAGAAAT CATCAGGAGG GTGGCATCTC CCCCTCCTCT

Siamang CCCTCCTCT

Orangutan GGACTCCCAG TGGAAGAAAT CATCAGGAGG GTGGCATCTC CCCCTCCTCT

Sumatran orangutan GGACTCCCAG TGGAAGAAAT CATCAGGAGG GTGGCATCTC CCCCTCCTCT

Chimpanzee GGACTCCCAG TGGAAGAAAT CATCAGGAGG GTGGCATCTC CCCCTCCTCT

Bonobo CCCNCCNCT

Human GGACTTCCAG TGGAAGAAAT CATCAGGAGG GTGGCATCTC CCCCTCCTCT

ex11 <-||-> ex12

2431 2441 2451 2461 2471

| | | | |

C R P L V S P D Q G P L E C I Q L

Rat GTGCCGGCCT CTGGTGTCCC CTGACCAGGG TCCCCTGGAG TGCATCCAGT

Mouse GTGCCGGCCA CTGGTGTCCC CTGACCAGGG TCCCCTCGAG TGTATCCAGT

Dog GTGCCGGCCC CGGGTGTCTC CTGACCATGG GCCACCCGAG TGTATCCAGC

Treeshrew GTGCCGGCCG ATGGTGTCCC CGGACCACGG GCCTCCAGAG TGCATCCAAC

Mouse lemur GTTCCGTCCG CTGGTGTCCC CGGACCACGG GCCACCTGAG TGCATCCAGC

Bushbaby GTGCCGGCCA CTGGTGTCCC CTGACCAGGG GCCACCCGAG TGCATCCAGC

Tarsier GTGCCGGTCA CTGGTGTCCC CTGACCACAG GCCACCCGAC TGCCTCCAGC

Drill GTGCTGGCCG CTGGTGACCC CTGATCACAG GCCACATGAG TGC-------

Macaque GTGCTGGCCG CTGGTGACCC CTGATCACAG GCCACATGAG TGC-------

Siamang GTGCCGGCCG CTGGTGACCC CTGATCACGG GCCACCTGAG TGTGTC-GGC

Orangutan GTGCCGGCCG CTGGTGACCC CTGATCACGG GCCGCCTGAG TGGGTC-GGC

Sumatran orangutan GTGCCGGCCC CTGGTGACCC CTGATCACGG GCCGCCTGAG TGGGTC-GGC

Chimpanzee GTGCCGGCCG CTGGTGACCC CTGATCACGG GCCGCCTGAG TGCATT-GGC

Bonobo GTGCCGGCCG CTGGTGACCC CTGATCACGG GCCGCCTGAG TGCATT-GGC

Human GTGCCGGCCG CTGGTGACCC CTGATCACGG GCCGCCTGAG TGCGTT-GGC

12A 12B

2481 2491 2501 2511 2521

| | | | |

M Q L C W E E A P D D R P S L D

Rat TGATGCAGCT GTGCTGGGAG GAAGCTCCAG ATGACAGGCC AAGCTTGGAC

Mouse TGATGCAGCT ATGCTGGGAG GAAGCTCCAG ATGACAGGCC AAGCTTGGAC

Dog TGATGGAGCA GTGCTGGGAG GAGGCTCCAG AGGACAGACC CAGCCTAGAC

Treeshrew TGATGAGGCA GTGCTGGGAG GAGGCCCCGG ATGACAGACC GAGCATGGAT

Mouse lemur TGATGAAGCA GTGCTGGGAG GAGGCTCCAG AGGACAGACC GAGCCTGGAC

Bushbaby TGATGAAGCA GTGCTGGGAG GAGGCTCCAG AGGACAGACC GAGCCTGGAC

Tarsier TGATGAAGCA GTGCTGGGAG GAGGTGCCAG AGGACAGACC AAGCCTGGAC

Drill ---TGAAGCA GTGCTGGGAG GA-GCTCCAG AGGACAGACC TA

Macaque ---TGAAGCA GTGCTGGGAG GA-GCTCCAG AGGACAGACC TAGCGTGGAC

Siamang TGATGAAGCA GTGCTGGGAC GAGGCTCCAG AGTACAGACC TA

Orangutan TGATGAAGCA GTGCTGGGAC GGGGCTCCAG AGGACAGACC TAGCATGGAC

Sumatran orangutan TGATGAAGCA GTGCTGGGAC GGGGCTCCAG AGGACAGACC TAGCATGGAC

Chimpanzee TGATGAAGAA GTGCTGGGAC GAGGCTCCAG AGGACAGACC TAGCATGGAC

Bonobo TGATGAAGAA GTGCTGGGAC GAGGCTCCAG AGGACAGACC TA

Human TGATGAAGAA GTGCTGGGAC GAGGCTCCAG AGGACAGACC TAGCATGGAC

12C

2531 2541 2551 2561 2571

| | | | |

Q I Y T Q F K S I N Q G K K T S V

Rat CAGATCTACA CACAGTTCAA AAGCATCAAC CAAGGCAAGA AGACAAGTGT

Mouse CAGATCTACA CACAGTTCAA AAGCATCAAC CAAGGCAAGA AGACAAGTGT

Dog CAGATCTACA CCCAGTTCAA AAGCATCAAT CAAGGCAAGA AGACCAGTGT

Treeshrew CAGATCTACA GCCAGTTCAA AAACATCAAT CAAGGCAAGA AAACCAGTGT

Mouse lemur CAGATCCACA CCCAGTTCAA AAGCATCACC CACGGCAAGA AGGCCAGCGT

Bushbaby CAGATCCACA CCCGGTTCAA AAGCATCAAC CAAGGCAAGA AGATCAGTGT

Tarsier CAGATCTACA CCCAGTTCTA AAGCATCAAC CAAGGCAAGA ---CCAGCGT

Macaque CAGATCTACA GCCAGTTCAA AAGCATCAAC CAAGGCAAGA AGACCAGTTT

Orangutan CAGATCTACA GCCAGTTCAA AAGCATCAAC CAAGGCAAGA AGACCAGTGT

Sumatran orangutan CAGATCTACA GCCAGTTCAA AAGCATCAAC CAAGGCAAGA AGACCAGTGT

Chimpanzee CAGATCTACA GCCAGTTCAA AAGCATCAAC CAAGGCAAGA GGACCAGTGT

Human CAGATCTACA GCCAGTTCAA AAGCATCAAC CAAGGCAAGA GGACCAGTGT

ex12 <-||-> ex13

13A

2581 2591 2601 2611 2621

| | | | |

A D S M L R M L E K Y S Q S L E G

Rat TGCTGACTCC ATGCTGCGGA TGCTGGAGAA GTATTCCCAA AGCCTGGAAG

Mouse TGTAGACTCC ATGCTGCGGA TGCTGGAGAA GTATTCCGAA AGCCTGGAAG

Dog TGCTGACTCT ATGCTATGGA TGCTAGAGAA GTATTCCCAG AACCTGGAGG

Treeshrew TGCTGACTCC ATGCTGCGGA TGCTGGAGAA GTATTCCCAG AACCTGGAAG

Mouse lemur AGCCGACTCT ATGCTGCGGA TGCTGGAGAC GTACTCGCGG CACCTGCAGG

Bushbaby TGCTGACTCC ATGCTGCGGA TGCTGGAAAA GTATTCCCAG AACCTGGAGG

Tarsier GGCTGACTCC ATGCTGCAGA TGTTGGAGAA GTACTCCCAG GACCTAGAGG

Macaque TGCTGACTCC ATGCTGCGGT TGCTGGAGAA ATATTCCCAG AACCTGGAGG

Orangutan TGCTGACTCC ATGCTGTAGT TGCTGGAGAA ATATTCCCAG AACCCAGAGG

Sumatran orangutan TGCTGACTCC ATGCTGTAGT TGCTGGAGAA ATATTCCCAG AACCCGGAGG

Chimpanzee TGCTGACTCC ATGCTGTGGT TGCTGGAGAA ATATTCCCAG AACCTGGAGG

Human TGCTGACTCC ATGCTGTGGT TGCTGGAGAA ATATTCCCAG AACCTGGAGG

13B

2631 2641 2651 2661 2671

| | | | |

L V Q E R T E E L E L E R R K T

Rat GTCTGGTCCA GGAGCGGACT GAGGAGCTGG AACTGGAGAG GCGGAAGACA

Mouse ATCTGGTCCA GGAGCGGACT GAGGAGCTGG AACTGGAGAG GCGGAAGACA

Dog ACCTGATCCA GGAGAGGACT GAGGAACTGG AGGTGGAGAA GCAGAAGACA

Treeshrew ACTTGGTCCA GGAGCGGACG GAGGAGCTGG AGCTGGAGAG ACAGAAGACT

Mouse lemur ACTTGGTCCA GGAGCGGACC GAGGAGCTGG AGCTGGAGAG GCAGAAGACA

Bushbaby ACTTGATCCA AGAGCGGACT GAGGAGCTGG AGCTGGAGAG ACAGAAGACA

Tarsier GTCTGATCTG GGAGTGGACT GAGGAGCTGG AGCTGGAAGG ACAGAAGACG

Macaque ACCTGATCCA GGAGCAGACT GAGGAACTGG AGCTGAAGAG AGAGAAGACA

Orangutan ACCTGATTCA GGAGCAGACT GAGGAACTGG AGCTGAAGAG AGAGAAGACA

Sumatran orangutan ACCTGATTCA GGAGCAGACT GAGGAACTGG AGCTGAAGAG AGAGAAGACA

Chimpanzee ACCTGATTCA GGAGCAGACT GAGGAACTGG AGCTGAAGAG AGAGAAGACA

Human ACCTGATTCA GGAGCAGACT GAGGAACTGG AGCTGAAGAG AGAGAAGACA

2681 2691 2701 2711 2721

| | | | |

E R L L S Q M L P P S V A H A L K

Rat GAGAGGCTGC TCTCACAGAT GCTCCCCCCG TCTGTCGCCC ATGCTCTAAA

Mouse GAGAGGCTGC TCTCGCAGAT GCTCCCTCCG TCTGTCGCCC ATGCTCTAAA

Dog GAGAGACTGC TCTCTCAGAT GCTCCCTTCG TCTGTGGCTG AAGCTCTGAA

Treeshrew GAAATGTTAC TGTCGCAGAT GCTTCCTCTG TCTGTGGCCG AAGCTCTGAA

Mouse lemur GAAAGGCTGC TCTCCCAGAT GCTCCCTCGA TCTGTGGCCC AAGCTCTGAA

Bushbaby GAAAGACTGC TCTCTCAGAT GCTCCCTCGA TCTGTGGCTG AAGCTCTGAA

Tarsier GAGAGGCTGC TCTGCCAGAT GCTGCCCCT

Macaque GAAAGGCTGC TCTGTCAGGT GATTCCCCTG TCTGTGGCCG ACGTTCTGAA

Orangutan GAAAGGCTGC TCTGTCAGAT GATTCCCCCG TCTGTGGCTG AAACTCTGAA

Sumatran orangutan GAAAGGCTGC TCTGTCAGAT GATTCCCCCG TCTGTGGCTG AAACTCTGAA

Chimpanzee GAAAGGCTGC TCTGTCAGAT GATTCCCCCG TCTGTGGCTG AAGCTCGGAA

Human GAAAGGCTGC TCTGTCAGAT GATTCCCCCG TCTGTGGCTG AAGCTCGGAA

ex13 <-||-> ex14

2731 2741 2751 2758 2766

| | | | |

M G T T V E P E Y F D Q V T I

Rat GATGGGAACA ACTGTGGAGC CC---GAGTA C--TTTGACC AAGTCACCAT

Mouse GATGGGGACA ACAGTGGAGC CC---GAGTA C--TTCGACC AGGTCACCAT

Dog AATGGGGGTA CCTGTGGAAC CA---GAGTA C--TTTGACC AGGTCACCAT

Treeshrew AATGGGGACA ACTGTGGAGC CA---GAGTA T--TTTGACC AGGTTACTAT

Mouse lemur AATGGGGGTG ACTGTGGAGC CG---GAGTA T--TTTGACC AGGTCACCAT

Bushbaby AATGGGGACG ACTGTGGAGC CA---GAGTA T--TTTGATC ATGTTACCAT

Macaque AATGGGGGTA ACTGTGGAAC CACAGGGGTA ACTGTGGAAC AGGTTACCAT

Orangutan AATGGGGGCA ACTGTGGAAC CA---GAGTA T--TTTGACC AGGTTACCAT

Sumatran orangutan AATGGGGGCA ACTGTGGAAC CA---GAGTA T--TTTGACC AGGTTACCAT

Chimpanzee AATGGGGGCA ACTGTGGAAC CA---GGGTA T--TTTGACC AGGTTACCAT

Human AATGGGGGCA ACTGTGGAAC CA---GGGTA T--TTTGACC AGGTTACCAT

14A 14B

2776 2786 2796 2806 2816

| | | | |

Y F S D I V G F T T I S A L S E P

Rat CTATTTCAGT GACATCGTGG GCTTTACCAC CATCTCAGCC TTGAGTGAGC

Mouse CTATTTCAGT GACATCGTGG GCTTCACCAC CATCTCAGCC TTGAGTGAGC

Dog ATACTTCAGT GACATTGTGG GCTTCACCAT CATCTCAGCC CTGAGTGAGC

Treeshrew ATACTTCAGT GACATTGTGG GTTTCACCAC CATCTCAGCC CTGAGTGAGC

Mouse lemur ATACTTCAGT GACATTGTGG GTTTTACCAC CATCTCAGCC CTGAGTGAAC

Bushbaby ATACTTCAGT GATATTGTGG GTTTCACTAC CATCTCAGCC CTAAGTGAAC

Macaque ATACTTCAGT AATATTGTAG GTTTCACCAT CATCTCAGCC CTGAGTGAAC

Orangutan ATACTTCAGT GACATTGTGG GTTTCACCAT CATCTCAGCC CTGAGTGAAC

Sumatran orangutan ATACTTCAGT GACATTGTGG GTTTCACCAT CATCTCAGCC CTGAGTGAAC

Chimpanzee ATACTTCAGT GACATTGTGG GTTTCACCAT CATCTCAGCC CTGAGTGAAC

Human ATACTTCAGT GACATTGTGG GTTTCACCAT CATCTCAGCC CTGAGTGAAC

2826 2836 2846 2856 2866

| | | | |

I E V V G F L N D L Y T M F D A

Rat CCATTGAGGT GGTGGGCTTC CTCAATGATC TCTACACGAT GTTTGATGCT

Mouse CCATTGAGGT GGTGGGCTTC CTCAATGATC TCTACACGCT GTTTGATGCT

Dog CCATTGAGGT GGTCGGCTTG CTCAACGACC TCTATACACT GTTCGATGCT

Treeshrew CCATCGAGGT GGTGGGCCTG CTCAATGACC TCTACACGCT GTTTGATGCT

Mouse lemur CCATTGAGGT GGTGGGCTTC CTCAACGATC TCTACACGCT GTTTGATGCT

Bushbaby CCATTGAGGT GGTGGGTTTG CTCAATGATC TCTACACACT GTTTGATGCT

Macaque CTATTGAGGT GGTGGGTTT- ---------- ----CATGCT GTTTGATGCT

Orangutan CCATTGAGGT GGTGGGCTTG CTCAACGATC TCTACATGCT GTTTGATGCT

Sumatran orangutan CCATTGAGGT GGCGGGCTTG CTCAACGATC TCTACATGCT GTTTGATGCT

Chimpanzee CCATTGAGGT GGTGGGCTTG CTCAACGATC TCTACATGCT GTTTGATGCT

Human CCATTGAGGC GGTGGGCTTG CTCAACGATC TCTACATGCT GTTTGATGCT

2876 2886 2896 2906 2916

| | | | |

V L D S H D V Y K V E T I G D A Y

Rat GTTCTAGACA GCCATGATGT GTATAAGGTA GAAACCATAG GGGATGCCTA

Mouse GTTCTCGACA GCCATGATGT GTATAAGGTG GAGACCATAG GGGATGCCTA

Dog GTTCTGGGCA GCCACGATGT GTATAAGGTG GAGACCATCG GGGATGCCTA

Treeshrew GTTCTGGGCA GCCATGATGT GTATAAG

Mouse lemur GTTTTGGGAA GCCATGATGT GTATAAGGTG GAGACCATCG GGGACGCCTA

Bushbaby GTTTTAAGAA ACCATGATGT GTATAAG

Tarsier GTG GAGACCGTCG GGGACGCCTA

Common marmoset GTG GAGACCATT- GGGATGCCTA

Macaque GTTCTGGGCA GCCATGACGT GTGTAAGGTG GAGACCACTG GGGATGCCTA

Orangutan GTTCTGGGCA GCCATGACCT GTATAAGGTG GAGACCATTG GGGACGCCTA

Sumatran orangutan GTTCTGGGCA GCCATGACCT GTATAAGGTG GAGACCATTG GGGACGCCTA

Chimpanzee GTTCTGGGCA GCCATGACCT GTATAAGGTG GAGACCACTG GGGACGCCTA

Human GTTCTGGGCA GCCATGACCT GTATAAGGTG GAGACCATTG GGGACGCCTA

ex14 <-||-> ex15 15A

2926 2936 2946 2956 2966

| | | | |

M V A S G L P R R N G N R H A A E

Rat CATGGTGGCA TCTGGGCTCC CTCGGCGCAA CGGAAATCGG CATGCTGCTG

Mouse CATGGTGGCT TCTGGTCTCC CTCGGCGCAA TGGAAATCGG CACGCTGCTG

Dog CATGGTGGCG TCGGGGCTGC CCCGGCGCAA CGGGAGCCGG CACGCGGCTG

Mouse lemur CATGGCGGTG TCGGGGCTGC CTCGGCGCAA CGGGAGCCGG CACGCGGCCG

Tarsier CAGGGTGGCC TCGGGACTGT CTTGGCGCAG CGGGAGCTGG CATGCAGCTG

Common marmoset CATGGTGGTG TCGGGGCTGC CTTGGTGCAA TGGAAGTTGG CATGTGGCTG

Macaque CATGATGGTG TCGGGGCTGC CTCAGCACAA TGGGAGTCAG CATGTCGCTG

Orangutan CATGGTGGTG TCGGGGCTGC CTCAGTGCAG TGGGAGTCAG CATGCGGCCA

Sumatran orangutan CATGGTGGTG TCGGGGCTGC CTCAGTGCAA TGGGAGTCAG CATGCGGCCA

Chimpanzee CATGGTGGTG TGGGGGCTGC CTCAGTGCAA TGGGAGTCAG CATGCGGCCG

Human CATGGTGGTG TGGGGGCTGC CTCAGTGCAA TGGCAGTCAG CATGCGGCCG

2976 2986 2996 3006 3016

| | | | |

I A N M A L E I L S Y A G N F R

Rat AGATTGCCAA CATGGCTCTG GAGATCCTTA GCTACGCAGG CAACTTCCGG

Mouse AGATCGCCAA CCTGGCCCTG GACATCCTTA GCTATGCAGG CAACTTTCGG

Dog AGATCGCCAA CATGGCCCTG GACATCCTTA GCTCTGTGAG AGGCTTCCGG

Mouse lemur AGATCGCCAA CATGGCCCTG GACGTCCTCA GCTCTGTGGG CGACTTCCGG

Tarsier AGGTCGCCAA CATGGCCCTG GACATCCCGA GCTCTGCAGG CGATTTCTGG

Common marmoset AGATCGCCA- ---------- -----CCTCA GCTCTGTGGG C-ACTTCTGG

Macaque AAATCAACAA CATGGCCCTG GATGTCCTCA GCTCTGTGGG TGACTTCTGG

Orangutan AGATC-ATAA CATGGCCCTG GATATCCTCG GCTCTGTGGT TGACTTCCGG

Sumatran orangutan AGATC-ATAA CATGGCCCTG GATATCCTCG GCTCTGTGGT TGACTTCCGG

Chimpanzee AGATCAATAA CATGGCTCTG GATATCCTTG GCTCTGTGGG TGACTTCTGG

Human AGATCAATAA CATGGCTCTG GATATCCTTG GCTCTGTGGG TGACTTCCGG

15B 15C 15D

3026 3036 3046 3056 3066

| | | | |

M R H A P D V P I R V R A G L H S

Rat ATGAGGCATG CACCTGATGT GCCCATCCGT GTCAGGGCCG GTCTGCATTC

Mouse ATGAGGCATG CACCCGATGT ACCCATCCGT GTCAGGGCAG GTCTGCATTC

Dog ATGAGGCATG CACCTGAGGT GCCTATTTAC ATCCGGGCTG GCCTGCACTC

Mouse lemur ATGAGGCACG CGCCCGACGT CCCCGTCCAC GTCAGGGCTG GCCTGCACTC

Tarsier ATGAGGCATG CACCCGACGT GCCTATTTGC ATCAGGGCTG GCCTGCATTC

Common marmoset ATGAGGCATG TGCCCAACGT TCCGGTTCAC ATCAGAGCTG GCCTGCATTC

Macaque ATGAGGCATG CACCCAACGT GCCCGTTTGC ATGAGGGCTG GCCTGCATTC

Orangutan AAGAGGCATG CACCCAACGT GCCTGTTTGC ATCAGGGCTG GCCTGCATTC

Sumatran orangutan AAGAGGCATG CACCCAACGT GCCTGTTTGC ATCAGGGCTG GCCTGCATTC

Chimpanzee AAGAGGCATG CACCCAACGT GCCCATTTGC ATCAGGGCTG GCCTGCATTC

Human AAGAGGCATG CACCCAACGT GCCCATTTGC ATCAGGGCTG GCCTGCATTC

3076 3086 3096 3106 3116

| | | | |

G P C V A G V V G L T M P R Y C L

Rat AGGGCCCTGT GTGGCAGGTG TTGTGGGTCT CACCATGCCT CGGTACTGCC

Mouse AGGACCCTGT GTGGCAGGTG TTGTGGGTCT CACCATGCCT CGGTACTGCC

Dog AGGGCCCTGC GTGGCAGGGG TCGTGGGCCT CACCATGCCG CGGTACTGCC

Treeshrew GGCCCTGT GTGGCAGGGG TCGTGGGTCT CACCATGCCT CGATACTGCC

Mouse lemur AGGGGCCTGT GTAGCAGGGG TCGTGGGTCT CCCGATGCCT CGGTACTGCC

Bushbaby GGCCCTGT GTAGCTGGGG TCGTGGGTCT CACGATGCCT CGGTATTGCC

Tarsier AG

Common marmoset AGGGACCTGT GCAGCAGGGG TCATGGGTCT CACGACGCCT CAGTATTGCC

Macaque AG

Orangutan AGGGCCCTGT GCAGCAGCGG TCGTGGGTCT CACGATGCCT TGGTATTGCC

Sumatran orangutan AGGGCCCTGT GCAGCAGCGG TCGTGGGTCT CACGATGCCT TGGTATTGCC

Chimpanzee AGGGCCCTGT GCAGCAGGGG TCGTGGGTCT CACGATGCCT TGGTATTGCC

Human AGGGCCCTGT GCAGCAGGGG TCGTGGGTCT CACGATGCCT TGGTATTGCC

ex15 <-||-> ex16

3126 3136 3146 3156 3166

| | | | |

F G D T V N T A S R M E S T G L

Rat TCTTTGGGGA CACCGTGAAC ACTGCTTCCA GGATGGAATC CACTGGACTG

Mouse TCTTTGGGGA CACCGTCAAC ACTGCCTCTA GGATGGAGTC CACTGGGCTG

Dog TCTTTGGTGA TACTGTTAAC ACTGCATCCC GGATGGAGTC CACAGGACTG

Treeshrew TCTTTGGGGA CACTGTCAAC ACTGCATCCC GGATGGAGTC CACAGGGCTG

Mouse lemur TCTTTGGGGA CACCGTCAAC ACTGCATCCC GGATGGAGTC CACGGGCCTG

Bushbaby TCTTTGGAGA TACTGTCAAC ACTGCATCCC GGATGGAGTC CACAGGGCTG

Common marmoset TCTTTGGGGA CAC------C ACGGCTTCCT GGATGGAGTC CACAGGGTTG

Orangutan TCTTTGGGGA CACTGTCAAC ACGGCTTCCT GGATGGAGTC CACAGGGCTG

Sumatran orangutan TCTTTGGGGA CACTGTCAAC ACGGCTTCCT GGATGGAGTC CACAGGGCTG

Chimpanzee TCTTTGGGTA CACTGTCAAC ACGGCTTCCC AGATGGAGTC CACAGGGCTG

Human TCTCTGGGGA CACTGTCAAC ACGGCTTCCC AGATGGAGTC CACAGGGCTG

3176 3186 3196 3206 3216

| | | | |

P Y R I H V S R N T V Q A L L S L

Rat CCATACAGAA TCCACGTCAG CCGAAACACT GTCCAGGCCC TGCTTAGCCT

Mouse CCTTACAGGA TCCATGTCAG TCAAAGCACT GTCCAGGCCC TGCTCAGCCT

Dog CCATACAGAA TTCACGTCAG CGGAAGCACA GCCCAGACAC TGCGCAGCCT

Treeshrew C

Mouse lemur C AA TTCACATCAG CCGAAGCACT ATGCAGACGC TGCTCAGCCT

Bushbaby CCATACAGAA TTCATACCAG CCGAAGCACT GTCCAGACAC TGCTCAGCCT

Tarsier CATACAGAA TTCATGTCAG CCAACGCACT GTCCAGACAC TGCTCAGCCT

Common marmoset CCATACAGCA TTCATGTCAG CCAAAGCACT CTCCAGATAC TGCTCAGTCC

Macaque CGTACAGAA TTCATGTCAG CCAAAGCACT GTCCAGATAC TGCTCAGCCT

Orangutan TCATACAGAA TTCATGTCAG CCGAAGCACT GTCCAGATAC TGCTTAGCCT

Sumatran orangutan TCATACAGAA TTCATGTCAG CCGAAGCACT GTCCAGATAC TGCTCAGCCT

Chimpanzee CCATACAGAA TTCATGTCAG CCGAAGCACT GTCCAGATAC TGCTCAGCCT

Human CCATACAGAA TTCATGTCAG CCAAAGCACT GTCCAGATAC TGCTCAGCCT

ex16 <-||-> ex17

3226 3236 3246 3256 3266

| | | | |

D E G Y K I D V R G Q T E L K G K

Rat TGACGAAGGC TACAAAATTG ATGTCAGAGG TCAGACCGAA CTGAAGGGGA

Mouse TGATGAAGGC TACAAAATTG ATGTCAGAGG TCAGACTGAA CTGAAGGGGA

Dog GGATGAAGGC TACAGAATTG ACATCAGAGG CCAGACTGAG CTAAAGGGGA

Treeshrew GGAA

Mouse lemur TGATGAAGGC TACAAAATTG ACATCAGAGG TCAGACTGAG CTGAAGGGCA

Bushbaby TGATAAAGGC TACAAAATTG ACATCAGAGG TCAGACTGAG CTGAAGGGGA

Tarsier TGATGAAGGT TACAAAATTG ACACCAGAGG TCAGACTGAG CTGAAGGGAA

Common marmoset TGA-GAAGGC TACCAAATTG ACATCAGAGG TCAGACGGAG CTGAAG

Macaque TGATGAAAGC TACCAAATTG ATAACAGAGG TCAGACTGAG CTGAAGGGGA

Orangutan TGATGAAAGC TACCAAATTG ATATCAGAGG TCAGACTGAG CTGAAGGGGA

Sumatran orangutan TGATGAAAGC TACCAAATTG ATATCAGAGG TCAGACTGAG CTGAAGGGGA

Chimpanzee TGATGAAAGC TACCAAATTG ATATCAGAGG TCAGACTGAG CTGCAGGGGA

Human TGATGAAAGC TACCAAATTG ATATCAGAGG TCAGACTGAG CTGAAGGGGA

17A ex17 <-||-> ex18

3276 3286 3296 3306 3316

| | | | |

G L E E T Y W L T G K T G F C R

Rat AGGGCTTGGA GGAGACTTAC TGGCTGACAG GGAAGACAGG ATTTTGCAGA

Mouse AGGGCTTGGA GGAGACCTAC TGGCTGACAG GGAAGGTAGG ATTCTGCAGA

Dog AGGGCGTAGA GGAGACCTAC TGGCTGGTGG GCAAGGCAGG CTTCCCTGGG

Treeshrew AGGGCATTGA GGAGACCTAC TGGCTGGCGG GGAAGGCAGG TTTCCCCAGA

Mouse lemur AAGGCGTGGA GGAGACCTAC TGGCTGGTGG GGAAGGCAGG CTTCCCCAGA

Bushbaby AGGGCATAGA GGAGACCTAC TGGCTGGTGG GAAAGGCAGG CTTCCCCAAA

Tarsier AGGGCATAGA GGAGACCTAC TGGCTGGTGG GGAAGGTGGG CTTCCCCAGG

Macaque AGGGCATCGA AGAGACCTAC TGGCTGCGGG GGAAGGCAGG TTTCCCCAGG

Orangutan AGGGCATCGA AGAGACCTAC TGGCTGGAGG GGAAGGCAGG CTTCCCCAGG

Sumatran orangutan AGGGCATCGA AGAGACCTAC TGGCTGGAGG GGAAGGCAGG CTTCCCCAGG

Chimpanzee AGGGCATCAA AGAGACCTAC TGGCTGG-GG GGAAGGCAGG CTTCCCCAGG

Human AGGGCATCGA AGAGACCTAC TGGGTGG-GG GGAAGGCAGG CTTCCCCAGG

18A

3326 3336 3346 3356 3366

| | | | |

S L P T P L S I Q P G D P W Q D H

Rat TCCCTCCCTA CACCTCTGTC CATCCAGCCT GGAGACCCAT GGCAGGACCA

Mouse CCCCTGCCTA CACCTCTGTC TATCAAGCCT GGAGACCCAT GGCAGGACCG

Dog TCCCTCCCCA CGCCTCTGGA CATCAAACCT GGGGATCCCT GGCAGGACCT

Treeshrew GCCCTCCCCA CCCCTCTGGA CATCAAGCCG GG

Mouse lemur CCCCTCCCCA AGCCCCTGGA CATCAAACCT GGAGAGCCCT GGCAGGACCT

Bushbaby CTCCTCCCCA CACCTCCGGA CATCAAACCT GG

Tarsier -CCCCCCCCA CACCTCCGGA CGTCAGTCTC GG

Common marmoset AGACCTTT GGCAAGACCT

Macaque CCCCTCCTCG CACCTGTGGA CATCAAACCT GGAGACCCTT GGTAAGACTT

Orangutan CCCCTCCCAG CACCTGTGGA CATCAAACCT GGAGACCCTT GGTAAGACTT

Sumatran orangutan CCCCTCCCAG CACCTGTGGA CATCAAACCT GG

Chimpanzee CCCCTCCCCG CACCTGTGGA CATCAGACCT GGAGACCCTT AGTAAGACTT

Human CCCCTCCCCG CACCTGTGGA CATCAGACCT GGAGACCCTT AGTAAGACTT

ex18 <-||-> ex19

18B 19A 19B

3376 3386 3396 3406 3415

| | | | |

Rat translated I N Q E I R T G F A K A r q s l a

Rat cDNA translated K L A R V C

Rat TATAAACCAA GAAATCCGGA CTGGCTTTGC CAAAGCTCGC CAGAGTCTGG

Rat cDNA TATAAACCAA GAAATCCGGA CTGGCTTTGC -AAAGCTCGC CAGAGTCTG-

Mouse CATAAACCAA GAAATCCGGA CTGGCTTTGC CAAAGCACGC CAGGGACTGG

Dog GATCAACCAA GAAATCAGGG TGGCCTTTGA CAAAGCACGC CAGAGCATGG

Mouse lemur CATAAACCAA GAAATCAAGG C

Common marmoset CATAAACCAT GAAATCCAGG TAGCCTTTAC C-AAGCCTGT CAGGACGCGG

Macaque CATAAACCAA GAAATCAAGG TGGCCTTTGC CAAAGCCTGT CAGGACACGG

Orangutan CATAAACCAA GAAATCAGGG CAGCCTTTGC CAAAGCCCGT CAGGACATGG

Chimpanzee CATAAACCAA GAAATCAGGG CAGACTTTGC CAAAGCCCGT CAGGACACGG

Human CATAAACCAA GAAATCAGGG CAGACTTTGC CAAAGCCCAT CAGGACACGG

19C

3424 3433 3443 3452 3461

| | | | |

Rat translated e p r s s g d t g p g p * *

Rat cDNA translated *

Rat CTGAGCCCAG GAGTTCAGGG GACACT-GGG CCAGGTCCCT GATAG

Rat cDNA CTGAGCC-AG GAGTTCAGGG GACACT-GGG CCAGGTCC-T GATAG

Mouse CTGAGCCCAG AAAATCAGGG GAGGCT-GGG CCAGGTCCCT GATAG

Dog CCAGGCCTGG GAGCTTGAGT AAGGCC-TTC GCTGGGCCCT GAGGA

Common marmoset CAGGGACCCA GAGCTCTGGA AAGGTCTCGG CCAGG-CCCT GCAGC

Macaque ---------- ---------- AAGACCTTGG CCAGG-CCCT GAGGA

Orangutan CAGAGACCCA GAGCTCCGGG AAGACCTCAG CCAGG-CCCT GAGGA

Chimpanzee CAGGGACCCA GAGCTCCGGG AAGACCTTGG CCAGG-CCCT GAGTA

Human CAGGGACCCA GAGCTCCGGG AAGAGCTTGG CCAGG-CCCT GAGTA

19D 19E 19F
