# Supplementary figures and images for: Degeneration of the Olfactory Guanylyl Cyclase D Gene during Primate Evolution
Source: PLoS One. 2007 Sep 12;2(9):e884. doi: 10.1371/journal.pone.0000884 (PMC1964805; doi:10.1371/journal.pone.0000884)

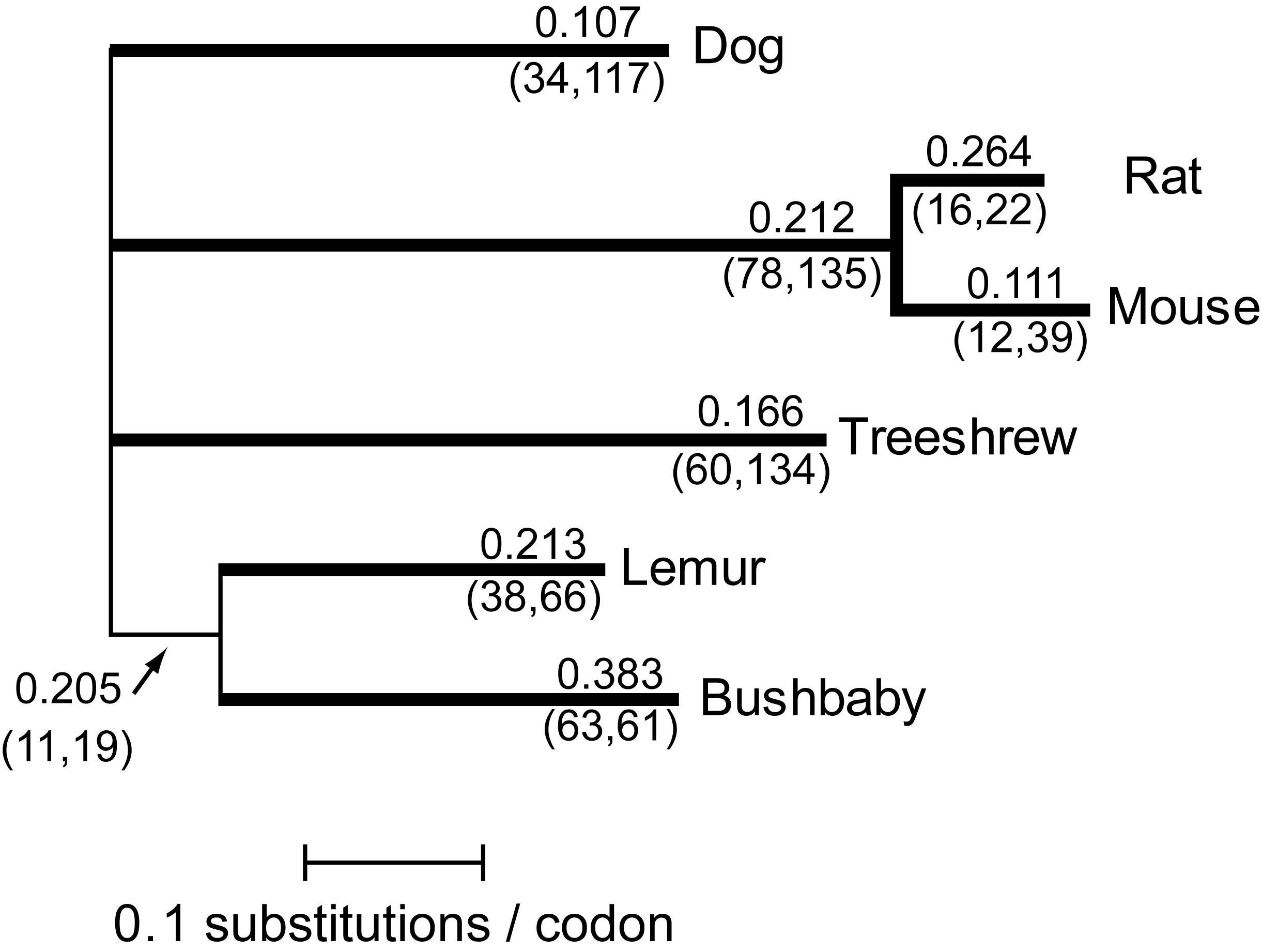

Supplement: Figure S2 — GC-D evolved under purifying selection in dog, rat, mouse, treeshrew, lemur and bushbaby. A phylogenetic tree of dog, rat, mouse, treeshrew, lemur, and bushbaby is shown: topology was taken from accepted species trees [7]–[10] and branch lengths represent an estimate of the total number of substitutions per codon in the GC-D sequences examined, as determined by PAML's codeml algorithm. We considered only a subset of species; if all species had been used, missing sequence data (exon deletions and/or absence from available data) would have meant that the number of codons available for analysis was too low. Nonsynonymous (Ka) and synonymous (Ks) rates of evolution were estimated for each branch using PAML's codeml (see methods, supplementary methods). The Ka/Ks ratio is given above each branch, and the number of non-synonymous and synonymous substitutions, respectively, are given below each branch in parentheses. For each branch of the tree, a statistical test was performed to determine whether the sequences observed are consistent with the null hypothesis of neutral evolution. Branches where the null (neutral) hypothesis was rejected with a Bonferroni-corrected p-value of 0.05 or less (i.e. branches where GC-D evolved under purifying selection) are drawn with thick lines. (4.52 MB DOC) [file pone.0000884.s005.doc]
