# Supplementary material for: Degeneration of the Olfactory Guanylyl Cyclase D Gene during Primate Evolution
Source: PLoS One. 2007 Sep 12;2(9):e884. doi: 10.1371/journal.pone.0000884 (PMC1964805; doi:10.1371/journal.pone.0000884)
Supplement: Text S1 — (0.06 MB DOC) [file pone.0000884.s006.doc]

**Text S1 Supporting Text**

## Degeneration of the Olfactory Guanylyl Cyclase D Gene During Primate Evolution

Janet M. Young, Hang Waters, Cora Dong, Hans-Jürgen Fülle, and Emily R. Liman

## PCR amplification of GC-D from primate DNA

Exons of GC-D were amplified using touch-down PCR with either Taq DNA Polymerase (exons 9, 10, 11, and 12) or Advantage 2 DNA Polymerase (exon 2). The Taq PCR reaction was performed in a total volume of 25 µl containing: 1 x PCR buffer (10 mM Tris-HCl, 50 mM KCl, 1.5 mM MgCl_2_ at pH 8.3), 0.2 mM dNTPs, 1-2 µM oligonucleotide primers, 1.25-2.5 U Taq DNA polymerase (NEB) and 10-20 ng genomic DNA. The touch-down amplification included 16 touch-down cycles, each consisting of 1 min at 94°C, 30 s at 64°C (decreasing 0.5°C /cycle) and 30 s at 72°C, followed by 40 amplification cycles, each consisting of 30 s at 94°C, 30 s at 66°C and 30 s at 72°C (increasing 2 s/cycle). Advantage 2 DNA Polymerase reactions were also performed in a volume of 25 µl, containing: 1 x SA PCR buffer (10 mM Tris-HCl, 50 mM KCl, 2 mM MgCl_2_ at pH 8.5), 0.2 mM dNTPs, 1-2 µM oligonucleotide primers, 10-20 ng DNA and 0.5 - 1 x mixture of TITANIUM Taq DNA Polymerase, proofreading polymerase and TaqStart Antibody (BD, Clontech) in the storage buffer. The PCR cycling was performed with 16 touch-down cycles of 30 s at 95°C, 30 s at 66°C (decreasing 0.5°C /cycle) and 1 min at 68°C, followed by 40 amplification cycles of 30 s at 95°C, 30 s at 58°C and 1 min at 68°C (increasing 2 s/cycle).

Following amplification, the PCR products were purified with QIAquick PCR Purification Kit (Qiagen) according to the manual and examined by gel electrophoresis for quality. The purified PCR products were sequenced in both directions with the original PCR primers, and sequence traces were analyzed with Sequencher and VectorNTI.

## Identification of GC-D from sequence databases

All available sequence traces from six primate species and treeshrew were obtained in fasta format from NCBI (ftp.ncbi.nih.gov) (see Supplementary Table 3). Blastable trace databases were created for each species using NCBI’s formatdb program, and the amino acid sequence of rat GC-D (AAC42057) was used in a sensitive tblastn search [1] of each database (E=10, v=1000, b=1000, -F F). Nucleotide sequences of exons 1 and 19 of human GC-D (the untranslated regions) were also used as blast queries. Chromatogram files were downloaded for any trace with even a weak match (E<10), as well as for any “mate-pairs” of matching chromatograms (sequences derived from the opposite end of the same genomic subclone) using custom perl scripts based on NCBI’s query_tracedb script.

Chromatograms were renamed based on name of the clone from which they derive using a custom perl script, so that pairs of sequences from the same clone could be recognized as mate pairs by Consed [2]. phredPhrap (www.phrap.org) was run on the resulting set of chromatograms to assemble overlapping sequences into contigs. The resulting contigs, along with singleton reads that failed to join any contig, were concatenated into one file. Amino acid sequences of all seven members of the guanylyl cyclase family (GC-A/P18910, GC-B/P16067, GC-C/P23897, GC-D/AAC42057, GC-E/P51840, GC-F/P51842, GC-G/P55205) were compared to the contigs-plus-singletons files using genewisedb [3], and any genomic sequence matching GC-D with a higher score than to any other guanylyl cyclase was used for further analysis as follows.

An artificially complete rat GC-D genomic sequence was constructed, using genomic sequence obtained from the rn4 genome assembly, with exon 3 from the published rat cDNA sequence (L37203) inserted into the assembly gap found between exons 2 and 4, and GC-D exon positions were annotated. Multipipmaker [4] was used to align this rat GC-D genomic sequence to GC-D-related contigs/singlets from each species, allowing identification, ordering, and orientation of the primate/treeshrew GC-D-containing sequences. Selected GC-D-containing contigs were extended by further rounds of trace database blast searches and phredPhrap assembly. Consed was then used to edit some poorly assembled GC-D-containing contigs, removing reads that contained a large number of discrepancies with the consensus. Genomic “scaffolds” (sets of ordered, oriented contigs) were then constructed, recruiting some additional contigs of interest based on either (a) mate-pair linkages to known GC-D containing contigs, as identified using Consed, or (b) extended similarity to dog GC-D genomic sequence, as identified by dotter comparisons [5] of all primate/treeshrew contigs and singletons to the dog GC-D genomic sequence. Comparisons with the dog GC-D genomic sequence helped to identify some GC-D-containing primate sequences, because dog still possesses all GC-D exons, and because dog has experienced much less intronic divergence from primate sequences than has rat, allowing most intronic sequences to be aligned and recognized.

GC-D genomic sequences were also identified from various genome assemblies (see Supplementary Table 3) using UCSC’s BLAT and Genome Browser (http://genome.ucsc.edu/). The rat GC-D cDNA sequence (L37203) was used as a BLAT query against each species’ genome, as well as two genes, CAPN5 and PKRIR, that map close to GC-D in rat. The presence of CAPN5 and PKRIR near the best match to rat GC-D in other species was used to confirm that the correct orthologous region had been identified, rather than a paralogous region such as the region containing GC-E.

These GC-D genomic sequences, and the scaffold sequences constructed from trace archive sequences, were aligned to the artificially complete rat GC-D genomic sequence using multipipmaker, and exon alignments were extracted from the resulting overall alignment using the program subalign [4]. A small number of additional exons or exon fragments that multipipmaker failed to align were added by hand. After adding sequences obtained from other primates by PCR, all exon alignments were concatenated to produce the final alignment shown in Supplementary Figure 1.

**Supplementary References**

1. Altschul SF, Madden TL, Schaffer AA, Zhang J, Zhang Z, et al. (1997) Gapped BLAST and PSI-BLAST: a new generation of protein database search programs. Nucleic Acids Res 25: 3389-3402.

2. Gordon D, Abajian C, Green P (1998) Consed: a graphical tool for sequence finishing. Genome Res 8: 195-202.

3. Birney E, Clamp M, Durbin R (2004) GeneWise and Genomewise. Genome Res 14: 988-995.

4. Schwartz S, Elnitski L, Li M, Weirauch M, Riemer C, et al. (2003) MultiPipMaker and supporting tools: Alignments and analysis of multiple genomic DNA sequences. Nucleic Acids Res 31: 3518-3524.

5. Sonnhammer EL, Durbin R (1995) A dot-matrix program with dynamic threshold control suited for genomic DNA and protein sequence analysis. Gene 167: GC1-10.

6. Fulle HJ, Vassar R, Foster DC, Yang RB, Axel R, et al. (1995) A receptor guanylyl cyclase expressed specifically in olfactory sensory neurons. Proc Natl Acad Sci U S A 92: 3571-3575.

7. Goodman M, Porter CA, Czelusniak J, Page SL, Schneider H, et al. (1998) Toward a phylogenetic classification of Primates based on DNA evidence complemented by fossil evidence. Mol Phylogenet Evol 9: 585-598.

8. Ray DA, Xing J, Hedges DJ, Hall MA, Laborde ME, et al. (2005) Alu insertion loci and platyrrhine primate phylogeny. Mol Phylogenet Evol 35: 117-126.

9. Opazo JC, Wildman DE, Prychitko T, Johnson RM, Goodman M (2006) Phylogenetic relationships and divergence times among New World monkeys (Platyrrhini, Primates). Mol Phylogenet Evol 40: 274-280.

10. Bininda-Emonds OR, Cardillo M, Jones KE, MacPhee RD, Beck RM, et al. (2007) The delayed rise of present-day mammals. Nature 446: 507-512.

**Legends to Supporting Figures**

**Figure S1. Alignment of GC-D nucleotide sequences from rat, mouse, dog, treeshrew, and multiple primate species.** Evolutionary changes that introduce a frameshift or stop codon that would severely disrupt the protein are highlighted in red; additional frameshifts or stop codons highlighted in gray might have more minimal effects on the protein. The predicted rat GC-D protein sequence is given above each block of the alignment, and below each block, exon boundaries and inactivating mutations are labeled (mutation labels correspond to Supplementary Table 2). Insertions/deletions are shown as “-“ characters; areas of missing sequence are entirely blank. Abbreviation: Red-backed squ monkey; red-backed squirrel monkey.

The rat cDNA sequence reported by Fülle *et al.* (L37203) [6] is also given for a small region of exon 2 and for exon 19. Compared to the rat genome assembly, L37203 has a 1-bp insertion and a nearby 1-bp deletion in exon 2, and several 1-bp deletions in exon 19, which together would subtly change the GC-D protein sequence. In all cases, the rat genome assembly sequence appears “correct”, in that it matches GC-D from other species – the discrepancies observed are therefore likely to represent either errors in the cDNA sequence, or polymorphic differences between the rat strain sequenced for the genome project (Brown Norway) and the rat strain from which the cDNA L37203 was derived (Sprague-Dawley).

**Figure S2. GC-D evolved under purifying selection in dog, rat, mouse, treeshrew, lemur and bushbaby.** A phylogenetic tree of dog, rat, mouse, treeshrew, lemur, and bushbaby is shown: topology was taken from accepted species trees [7-10] and branch lengths represent an estimate of the total number of substitutions per codon in the GC-D sequences examined, as determined by PAML’s codeml algorithm. We considered only a subset of species; if all species had been used, missing sequence data (exon deletions and/or absence from available data) would have meant that the number of codons available for analysis was too low. Nonsynonymous (*K_a_*) and synonymous (*K_s_*) rates of evolution were estimated for each branch using PAML’s codeml (see methods, supplementary methods). The *K_a_/K_s_* ratio is given above each branch, and the number of non-synonymous and synonymous substitutions, respectively, are given below each branch in parentheses. For each branch of the tree, a statistical test was performed to determine whether the sequences observed are consistent with the null hypothesis of neutral evolution. Branches where the null (neutral) hypothesis was rejected with a Bonferroni-corrected p-value of 0.05 or less (i.e. branches where GC-D evolved under purifying selection) are drawn with thick lines.
