# Supplementary material for: Degeneration of the Olfactory Guanylyl Cyclase D Gene during Primate Evolution
Source: PLoS One. 2007 Sep 12;2(9):e884. doi: 10.1371/journal.pone.0000884 (PMC1964805; doi:10.1371/journal.pone.0000884)
Supplement: Supplementary file 6 [file pone.0000884.s002.doc]

			Exon	2																	3			4	5	6	7	8	9						10											11						12			13		14		15				#####	#####	18		1		
			Mutation code	A	B	C	D	E	F	G	H	I	J	K	L	M	N	O	P	Q	A	B	C	A	A	A	A	A	A	B	C	D	E	F	A	B	C	D	E	F	G	H	I	J	K	A	B	C	D	E	F	A	B	C	A	B	A	B	A	B	C	D	A	A	A	B	A	B	C
	Species	Data Type	Scientific name	Exon d	136i	184in	201d	213d	254d	307d	374	420d	460i	544d	590	640d	735d	775i	813d	830d	871d	924i	1069	Exon d	Exon d	1629	1790	1859	1936	1958	1965d	2008	2009	2064	2102	2132	2140	2142	2172	2204	2207	2211	2216	2229	2237	2246	2294	2297	2327	2359	2367	2474d	2477	2503	2549	2597	2756	2758i	2915	2981	2985d	3017	Exon del	3229de	3303	3326	3365	3368	3406
	Tarsier	2	*Tarsius syrichta*	-	X	-	-	-	-	-	.	.	.	.	.	?	?	?	?	.	.	.	.	?	?	-	-	.	.	.	.	.	.	.	-	-	-	-	-	-	-	-	X	-	-	X	-	-	-	-	X	-	-	-	X	-	.	.	-	-	-	-	.	-	-	X	.	.	.
New World monk	Titi	3	*Callicebus moloch*	-	-	-	-	-	-	-	X	X	-	-	-	X	X	X	X	-	.	.	.	?	?	.	.	.	.	?	.	?	.	?	.	X	-	-	X	-	-	-	-	-	-	-	-	-	-	.	.	.	?	.	.	.	.	.	?	.	?	?	.	?	.	.	.	.	?
	Owl monkey	3	*Aotus azarai*	.	.	+	.	+	+	?	.	.	.	?	+	+	+	+	+	.	.	.	.	?	?	.	.	.	.	?	.	?	.	?	.	-	-	-	-	-	-	-	-	X	-	-	-	-	-	.	.	.	?	.	.	.	.	.	?	.	?	?	.	?	.	.	.	.	?
	Red-backed squirrel mo	3	*Saimiri oerstedii*	-	-	X	-	X	X	X	-	-	-	X	X	X	X	X	X	-	.	.	.	?	?	.	.	.	.	?	.	?	.	?	.	-	-	-	-	-	-	X	-	X	-	-	X	-	-	.	.	.	?	.	.	.	.	.	?	.	?	?	.	?	.	.	.	.	?
	Common squirrel monk	3	*Saimiri sciureus*	.	.	+	.	+	+	?	.	.	.	?	+	+	+	+	+	.	.	.	.	?	?	.	.	.	.	?	.	?	.	?	.	-	-	-	-	-	-	-	-	X	-	-	X	-	-	.	.	.	?	.	.	.	.	.	?	.	?	?	.	?	.	.	.	.	?
	Common marmoset	2	*Callithrix jacchus*	-	-	X	-	X	X	-	-	-	X	-	X	X	X	X	X	X	.	.	.	?	?	.	.	.	-	X	-	X	-	X	-	-	-	-	-	-	-	-	-	X	-	X	-	-	-	-	-	.	?	.	.	.	.	.	X	-	X	X	-	X	.	.	-	-	X
	Pygmy marmoset	3	*Callithrix pygmaea*	-	-	X	-	X	X	-	-	-	X	-	X	X	X	X	X	X	.	.	.	?	?	.	.	.	.	?	.	?	.	?	.	-	-	-	-	-	-	-	-	X	-	X	-	-	-	.	.	.	?	.	.	.	.	.	?	.	?	?	.	?	.	.	.	.	?
	Spider monkey	3	*Ateles geoffroyi*	-	-	-	-	-	X	-	-	-	-	-	-	X	X	X	X	-	.	.	.	?	?	.	.	.	.	?	.	?	.	?	.	.	.	.	.	.	.	.	.	?	.	.	.	.	.	.	.	.	?	.	.	.	.	.	?	.	?	?	.	?	.	.	.	.	?
	Howler monkey	3	*Alouatta seniculus*	-	-	-	X	-	X	-	-	-	-	-	X	X	X	X	X	-	.	.	.	?	?	.	.	.	.	?	.	?	.	?	.	.	.	.	.	.	.	.	.	?	.	.	.	.	.	.	.	.	?	.	.	.	.	.	?	.	?	?	.	?	.	.	.	.	?
Catarrhine prima	Drill	3	*Mandrillus leucopha*	+	.	.	.	.	.	.	.	.	.	.	.	?	?	?	?	.	?	-	?	+	+	?	?	?	X	-	-	-	-	-	?	-	-	-	-	-	-	-	-	-	X	-	-	X	-	-	.	X	?	X	.	.	?	?	.	.	.	.	?	.	.	.	.	+	.
	Rhesus macaque	1	*Macaca mulatta*	X	.	.	.	.	.	.	.	.	.	.	.	?	?	?	?	.	X	-	X	X	X	X	X	X	X	-	-	-	-	-	X	-	-	-	-	-	X	-	-	-	X	-	-	-	-	-	-	X	?	X	-	-	X	X	-	-	-	-	X	-	-	-	-	X	-
	Siamang	3	*Hylobates syndactylu*	+	.	.	.	.	.	.	.	.	.	.	.	?	?	?	?	.	.	.	.	+	+	.	.	.	.	-	X	-	-	-	.	-	X	.	-	X	-	-	-	-	-	.	-	-	-	-	.	-	X	-	.	.	.	.	.	.	.	.	.	.	.	.	.	+	.
	Orangutan	2	*Pongo pygmaeus*	+	.	.	.	.	.	.	.	.	.	.	.	?	?	?	?	.	-	-	-	+	+	-	-	-	-	-	X	-	-	-	-	-	-	X	-	-	-	-	-	-	-	-	-	-	X	-	-	-	X	-	-	X	-	-	-	X	-	-	-	-	-	-	-	X	-
	Sumatran orangutan	2/3	*Pongo pygmaeus abe*	+	.	.	.	.	.	.	.	.	.	.	.	?	?	?	?	.	-	-	-	+	+	.	-	.	-	-	X	-	-	-	-	-	-	X	-	-	-	-	-	-	-	-	-	-	X	-	-	-	X	-	-	X	-	-	-	X	-	-	-	-	-	-	.	+	.
	Gorilla	3	*Gorilla gorilla*	+	.	.	.	.	.	.	.	.	.	.	.	?	?	?	?	.	.	-	.	+	+	.	.	.	-	-	X	-	-	.	.	-	-	-	-	X	-	-	-	-	-	-	.	.	.	.	.	.	+	.	.	.	.	.	.	.	.	.	.	.	?	.	?	+	.
	Chimpanzee	1	*Pan troglodytes*	X	.	.	.	.	.	.	.	.	.	.	.	?	?	?	?	.	-	X	-	X	X	-	-	-	-	-	X	-	-	-	-	-	-	-	-	X	-	-	-	-	-	-	-	-	-	-	-	-	X	-	-	-	-	-	-	-	-	-	-	-	X	-	X	X	-
	Bonobo	3	*Pan paniscus*	+	.	.	.	.	.	.	.	.	.	.	.	?	?	?	?	.	.	X	.	+	+	.	.	.	-	-	X	-	X	-	.	-	-	-	-	X	-	-	-	-	-	-	-	-	-	-	.	-	X	-	.	.	.	.	.	.	.	.	.	.	+	.	+	+	.
	Human	1	*Homo sapiens*	X	.	.	.	.	.	.	.	.	.	.	.	?	?	?	?	.	-	X	-	X	X	-	-	-	-	-	X	-	-	-	-	-	-	-	-	X	-	-	-	-	-	-	-	-	-	X	-	-	X	-	-	-	-	-	-	-	-	-	-	-	X	-	X	X	-

Data types:
1: Genome Assembly
2: Trace archive
3: PCR and sequencing

- \- Sequence available, mutation not present
- . Sequence not available, inferred from tree that mutation is probably not present
- X Sequence available, mutation present
- \+ Sequence not available, inferred from tree that mutation is probably present
- ? Sequence not available, cannot infer whether mutation is present

9			Numbe r of mutatio ns observe d*	Number of mutatio ns inferred	Minimu m number total mutation s (observe d plus
D	E	F			
3424d	3448	3457			
.	?	?	6	0	6
.	+	+	8	0	8
.	+	+	1	8	9
.	+	+	13	0	13
.	+	+	2	8	10
-	X	X	19	0	19
.	+	+	12	0	12
.	+	+	5	0	5
.	+	+	7	0	7
?	+	+	5	3	8
X	X	X	17	0	17
.	+	+	4	3	7
-	X	X	6	3	9
.	+	+	6	3	9
.	+	+	2	4	6
-	X	X	8	0	8
.	+	+	5	4	9
-	X	X	9	0	9

*Number of inactivating mutations observed, not including exon 19 mutations that probably have minimal effect on
